# Ionic Highways under Multivariate‐Coupled Strategies: Ultrahigh Power Generation from Industrial Waste Liquors Using Robust COF Membranes

**DOI:** 10.1002/advs.202523044

**Published:** 2026-01-09

**Authors:** Hongyan Qi, Weibo Sun, Jundong Zhong, Zhen Xing, Tingting Xu, Shuang Zhao, Haoyang Tan, Ziyi Hu, Hujun Qian, Yuchen Wu, Haibo Zhang, Jianxin Mu, Xuanbo Zhu, Lei Jiang

**Affiliations:** ^1^ National and Local Joint Engineering Laboratory for Synthetic Technology of High‐Performance Polymer College of Chemistry Jilin University Changchun China; ^2^ State Key Laboratory of Supramolecular Structure and Materials Institute of Theoretical Chemistry College of Chemistry Jilin University Changchun China; ^3^ International Center of Future Science College of Chemistry Jilin University Changchun China; ^4^ CAS Key Laboratory of Bio‐inspired Materials and Interfacial Science Technical Institute of Physics and Chemistry Chinese Academy of Sciences Beijing China

**Keywords:** covalent organic framework membrane, multivariate‐coupled, osmotic energy harvesting, smart response device

## Abstract

Efficiently harvesting the intrinsic energy from low‐grade heat, acidity, and high salinity of desulfurization waste liquors is crucial for sustainable management, yet remains challenging due to the instability of conventional membranes under such extreme multi‐physics conditions. Herein, we report a high‐performance osmotic energy conversion device engineered via a multivariate coupling strategy. The core of this device is a robust membrane based on β‐ketoenamine‐linked covalent organic frameworks (COFs), featuring nanochannels functionalized with tailored stimuli‐responsive groups to dynamically regulate surface charge density. The β‐ketoenamine linkage endows exceptional membrane stability, enabling durable operation under coupled thermal, chemical, and electrochemical stresses. Experimental and computational studies demonstrate that the remarkable power enhancement stems not only from acid‐induced protonation that boosts charge density, but also from the utilization of low‐grade heat to accelerate ion transport. By simulating the multi‐physical field coupling in real waste liquors, the device achieves an ultrahigh power output of 258.81 W m^−^^2^, surpassing commercial benchmarks by 52‐fold. This COF membrane, with its exceptional permeability, selectivity, and stability, paves the way for high‐efficiency energy harvesting from hostile industrial environments.

## Graphical abstract

This study presents a multivariate‐coupled strategy to fabricate TB‐COF membranes with tunable charge density via uniformly distributed Bpy groups, enabling efficient ion transport and energy harvesting. The robust β‐ketoenamine‐linked structure ensures stability, achieving power outputs of 53.08 W m^−2^ (pH 4), 190.52 W m^−2^ (low‐grade heat), and 258.81 W m^−2^ (acid / heat). The designed membranes exhibit exceptional ion selectivity and permeability, enabling high‐performance nanofluidic energy harvesting devices.

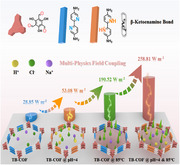

## 1 Introduction

The current energy landscape presents a diverse array of underutilized renewable sources, particularly within aqueous environments, which harbor abundant and sustainable reserves in the form of low‐grade heat (≤100°C) and waste acids for energy harvesting [1, 2]. However, current energy harvesting methods and materials remain inadequate, particularly in adapting to complex real‐world conditions. For example, desulfurization waste liquid from thermal power plants, especially from wet flue gas desulfurization, contains high salinity (0.8‐2.6 mol L^−1^ Cl^−^) and acidic pH (4–6), resulting in strong corrosivity that challenges material stability. Repurposing such energy under extreme conditions is essential to reduce environmental pollution [3, 4]. The coupling of multiple physical fields is a very suitable strategy, which has been found to provide potential optimization solutions for device design and ensure improved application performance [5, 6]. Thus, there is a critical need to develop materials resistant to solvents, high temperature, and acidic/alkaline conditions to ensure device reliability [7, 8, 9, 10]. The inspiration comes from the biological ion channels that dynamically regulate transmembrane ion transport through stimulus‐responsive gating. The current artificial nanofluid systems mainly utilize physical fields such as light, pressure, temperature, pH, and chemistry to improve ion transport behavior [7, 11, 12, 13]. Rational material design and long‐term operational stability are equally crucial [14, 15, 16, 17, 18, 19, 20]. Membrane materials, in particular, play a key role in enabling stable energy harvesting across applications, yet the relationship between their internal mechanisms and energy efficiency requires deeper investigation [18, 21, 22, 23, 24, 25, 26, 27].

COFs are crystalline porous materials constructed from covalently linked monomers, forming predictable two‐dimensional (2D) or three‐dimensional (3D) structures [28, 29, 30]. Their high crystallinity, large surface area, tunable porosity, and tailored chemical environments have led to broad applications in adsorption, separation, catalysis, energy storage, and conversion [7, 16, 29, 31, 32]. However, the stability of COFs under harsh conditions, such as strong acids, bases, or oxidizing and reducing environments, remains a challenge, limiting their applications and raises the requirements for COF materials [8, 22, 33]. Currently, COFs based on the β‐ketoenamine structure are among the more stable types of imine COFs reported [34, 35]. These COFs form initially through a reversible Schiff base reaction, followed by an irreversible transformation into a ketoenamine‐linked framework while maintaining crystallinity [36, 37]. This irreversible bonding enhances stability in demanding environments including boiling water, strong acids, and alkaline solutions [24, 38, 39]. Reasonable modular COF design has begun to be utilized in the process of energy conversion and harvesting, adapting to diverse environments through purposeful design [40, 41]. Therefore, it is essential to develop technologies that can address the energy conversion requirements for waste energy sources, including low‐grade heat, waste acids, and high‐salinity solutions, while demonstrating their practicality in the collection of osmotic energy [42, 43].

Herein, we synthesized a free‐standing, flexible, and porous β‐ketoenamine COF membrane at room temperature for the conversion and collection of osmotic energy in a multi‐responsive environment. In this study, we prepared a COF (TB‐COF) moiety connected by β‐ketoenamine and introduced 2,2'‐bipyridine‐5,5'‐diamine (Bpy) intelligent functional groups to enhance its applicability in multi‐scenario coupling [44, 45]. First, the induced electron interactions between TB‐COF and π‐π facilitate the collection of osmotic energy that significantly exceeds commercial standards under specific salt concentration gradients. Secondly, in acidic environments, the Bpy functional groups undergo efficient protonation, increasing nanochannel charge density and enhancing transmembrane ion transport. Furthermore, the ultra‐stable β‐ketoenamine linkage enables effective utilization of low‐grade heat, promoting thermal acceleration of ion migration. Finally, under integrated multi‐physical conditions simulating the zero‐discharge scenario of desulfurization wastewater from thermal power plants, the TB‐COF membrane achieves ultrahigh‐power output of 258.81 W m^−^^2^, significantly exceeding the initial value of 28.85 W m^−^^2^, and demonstrates ultra‐stable osmotic energy conversion. The multivariate coupling strategy developed in this study enables efficient, rational, and sufficient energy harvesting from waste sources, highlighting the great potential of the designed membrane for practical osmotic energy conversion.

## 2 Results and Discussion

### 2.1 Design Principles and Working Mechanisms

**FIGURE 1:**
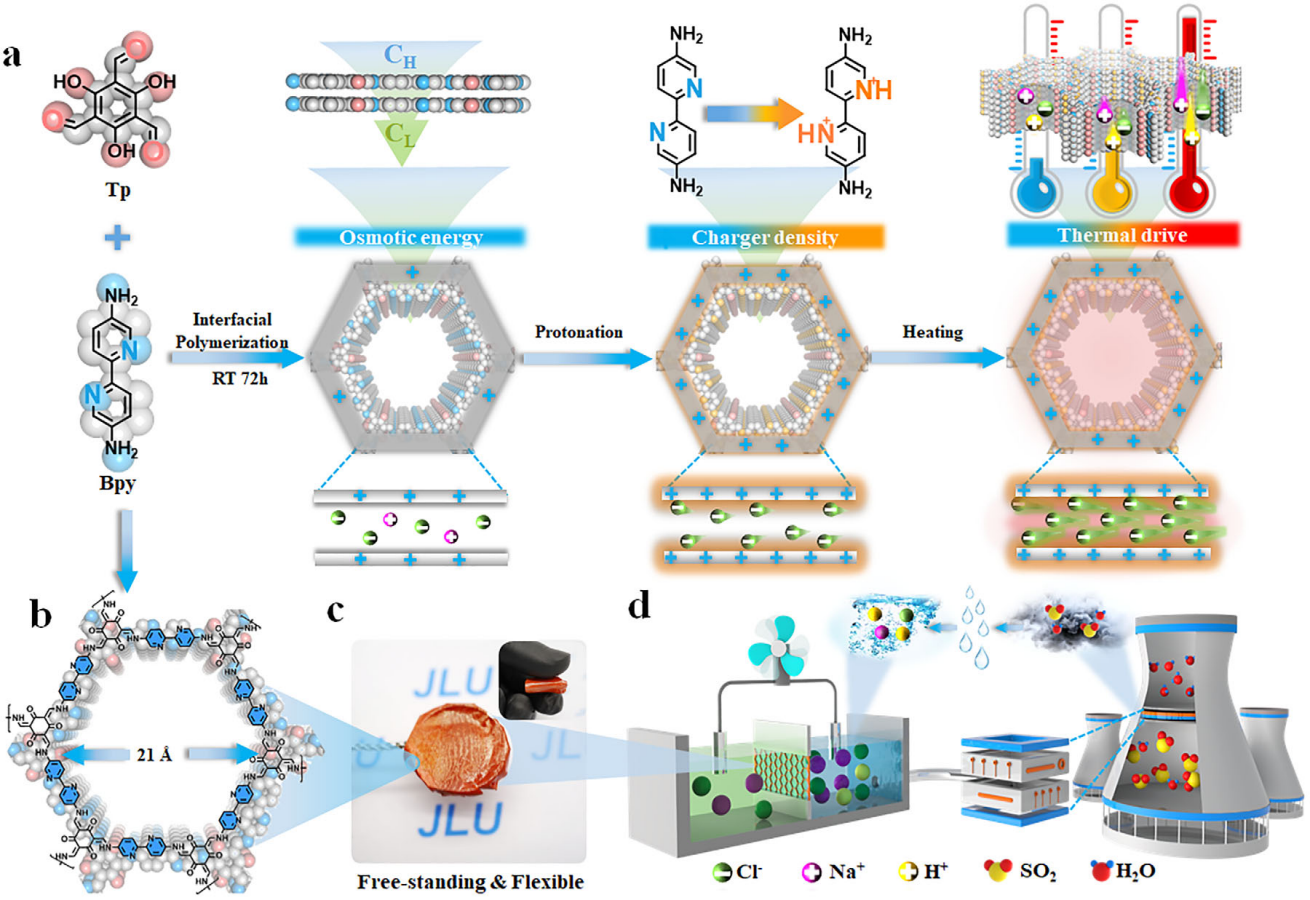
Construction of the Intelligent Response Membrane and the Working Mechanism of Efficient Osmotic Energy Harvesting. (a) The TB‐COF film was prepared through interfacial polymerization of Tp and Bpy monomers in a two‐phase solvent. The responsive monomer Bpy can undergo efficient protonation at pH 4, which enhances the charge density within the nanochannel. Additionally, utilizing low‐grade heat sources to accelerate the transport of ions in nano‐confined channels can further improve the efficiency of osmotic energy harvesting. (b) Top views of the eclipsed AA stacking model simulate a schematic representation of the one‐dimensional channel of TB‐COF through which ions can pass, with the size of the nanochannel measuring 21 Å. (c) The TB‐COF film, obtained through interface aggregation, exhibits free‐standing and flexible properties, enabling it to better adapt to various working environments and facilitate the preparation of energy harvesting devices. (d) Establishing a Neterfo‐based extreme separation system allows for the treatment and response to high salinity salt ions, acidic environments, and low‐grade waste heat (accelerated ion transmembrane transport) in desulfurization wastewater. The TB‐COF membrane achieves efficient osmotic energy output between two electrolytic cells.

To design devices for environmental remediation and energy recovery, we adopt a multivariate‐coupled strategy that incorporates targeted functional groups into β‐ketoenamine‐linked COFs. This design enhances adaptability to harsh environments, such as high‐salinity solutions, acidic waste streams, and low‐grade heat, while enabling efficient and stable osmotic energy harvesting. The design and synthesis of nanofluidic osmotic energy generators must meet the following criteria: (i) sustained ion transport under specific environmental conditions; (ii) nanoconfined channels with responsive functional groups to modulate surface charge and ensure high ion selectivity and permeability; and (iii) a scalable membrane fabrication route suitable for environmental and energy applications. Using interfacial polymerization in a two‐phase system, we successfully integrated functionalized monomers into stable β‐ketoenamine COFs. Specifically, a TB‐COF membrane was synthesized by reacting Disperse Bpy and Tp (1,3,5‐triformylphloroglucinol) at room temperature, yielding ordered nanopores (Figure 1a). Stable harvesting of osmotic energy can be achieved in environments such as high‐salinity solutions (osmotic energy), waste acid solutions (charge density regulation), and low‐grade heat (accelerated ion transmembrane transport).

Top views of the eclipsed AA stacking model are provided in Figure 1b, illustrating the one‐dimensional nanochannels of TB‐COF that facilitate ion transport. These open channels have a diameter of 21 Å, enabling unimpeded ion permeation. As shown in Figure 1c, the membrane exhibits free‐standing and flexible characteristics, enhancing its adaptability and suitability for diverse operational environments, and simplifying integration into energy harvesting devices. The operating principle of these devices resembles that of an “ultrafiltration membrane”, a biological membrane capable of harvesting osmotic energy through selective screening, separation, and recognition processes [46, 47]. As depicted in Figure 1d and Scheme S1, high‐salinity wastewater, acidic solutions, and low‐grade heat generated from thermal power plant desulfurization can be captured via conventional wet desulfurization. By directing this wastewater through a tailored osmotic energy harvesting device, the multivariate‐coupled TB‐COF membrane plays a critical role in recovering sustainable energy from waste sources, thereby simultaneously reducing environmental pollution and alleviating energy shortages.

### 2.2 Characterization of the Basic Structure of TB‐COF Membranes

**FIGURE 2:**
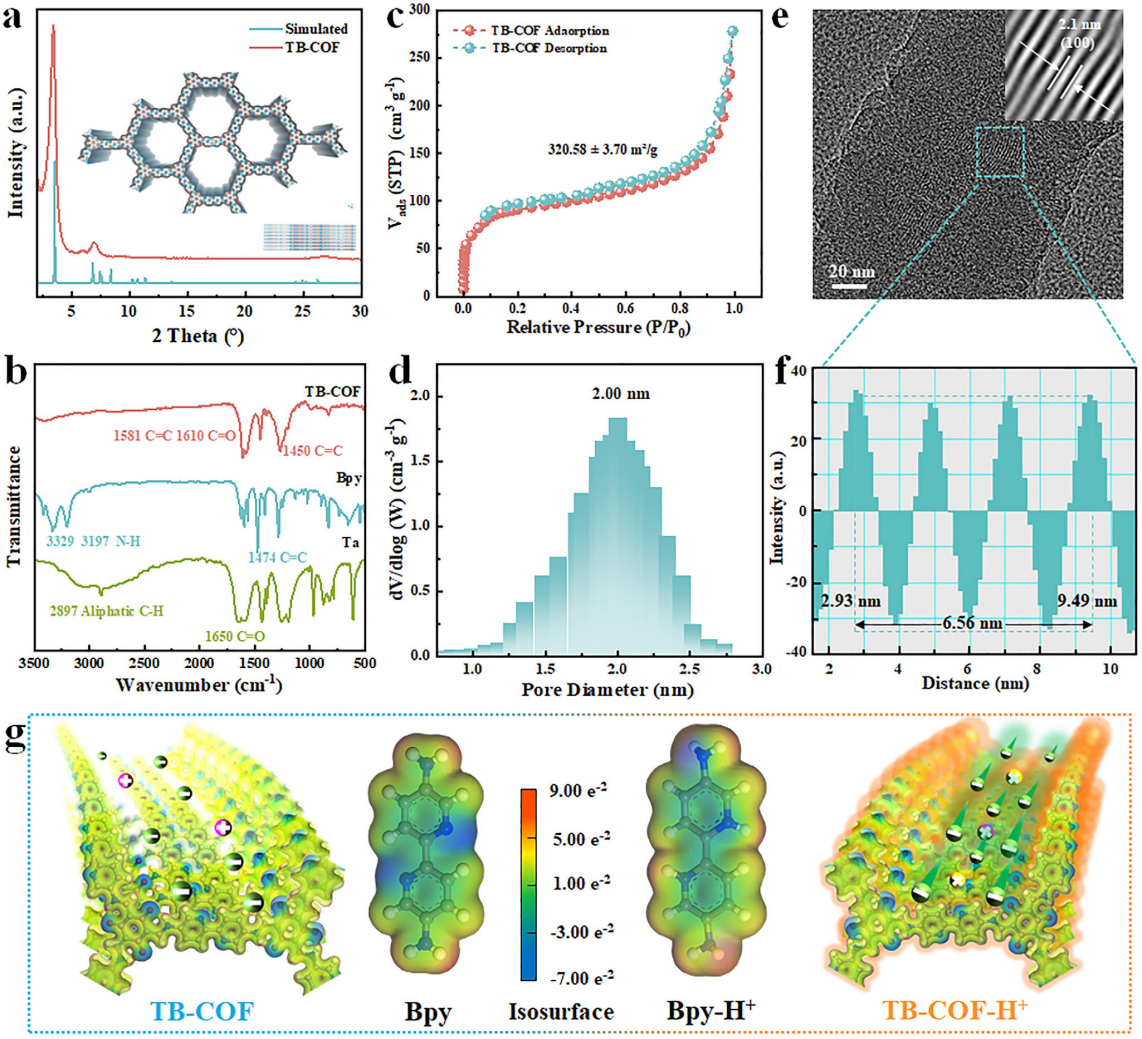
Characterization of the Basic Structure of the TB‐COF Membrane. (a) XRD spectra of TB‐COF, along with simulated XRD curves for this topology, indicate that the membrane structure resulting from interfacial polymerization maintains good crystallinity. (b) The FT‐IR spectrum of TB‐COF and its corresponding monomers shows the generation of the C═C keto‐enamine linkage, while the characteristic peaks of the alpha C─H of the Tp monomer and the N─H of the Bpy monomer have disappeared, confirming the successful synthesis of TB‐COF. (c) The N_2_ adsorption and desorption isotherms of TB‐COF demonstrate rapid gas adsorption in the range of 0.0 to 0.1 (P/P_0_), indicating that TB‐COF possesses a significant number of microporous structures in a highly crystalline state. (d) The pore size distribution of TB‐COF was calculated using the NLDFT cylindrical pore model. (e) The HRTEM image of TB‐COF displays clear and distinct diffraction patterns. The 2.1 nm wide lattice fringes correspond to the simulated unit cell size (100). (f) Distance versus intensity plots were obtained from two rectangular sections measuring 2.1 nm in (e). (g) The ESP of Bpy and Bpy‐H^+^ monomer structures. The ESP results indicate that the maximum positive charge (red area) is distributed within the pores formed by the Bpy‐H^+^ functional group compared to Bpy. It can be observed that there are active Cl^−^ accommodation sites in Bpy‐H^+^, surrounded by strong electronegative O/N atoms and activation sites.

X‐ray diffraction (XRD) analysis confirmed the high crystallinity of the TB‐COF membranes (Figure 2a). The pattern showed a strong peak at 3.6° corresponding to the (100) plane and a broad peak at 27.1° attributed to the (001) plane, matching the simulated AA‐stacking mode (blue line), which is thermodynamically favorable [37, 48]. The chemical structure was verified by Fourier transform infrared (FT‐IR) spectroscopy (Figure 2b) and solid‐state ^1^ ^3^C NMR (Figure S1). The FT‐IR spectra displayed a C═C stretch at 1581 cm^−1^, along with the disappearance of the Tp aldehyde peak (2897 cm^−1^) and Bpy amine peaks (3329 and 3197 cm^−1^), confirming successful formation of the β‐ketoenamine linkage [49, 50]. The solid‐state NMR spectra of TB‐COF exhibit resonances at 150.6 and 107.4 ppm, corresponding to the enamine carbon and the α‐enamine carbon. To further validate the reliability of the material preparation, we also conducted stability tests on the material. Thermal gravimetric analysis (TGA) revealed that the TB‐COF membranes were stable up to 400°C, suggesting that the membranes had high thermal stability (Figure S2). Thus, TB‐COF membranes could meet the requirements of most of the practical applications. Here, nitrogen gas (N_2_) was utilized as a probe to investigate the pore structure and microenvironment of TB‐COF. The N_2_ adsorption‐desorption isotherms at 77 K are shown in Figure 2c, and the Brunauer‐Emmett‐Teller (BET) surface area was 320.58 m^2^ g^−1^, and rapid N_2_ uptake at low pressure (P/P_0_< 0.1) indicated abundant nanopores and high crystallinity. The regular nanochannels formed create an optimal environment for ion transport. The pore size distribution derived from the Nonlinear Density Functional Theory (NLDFT) analysis showed a sharp peak at 2.0 nm (Figure 2d), consistent with the structural model, confirming well‐defined nanochannels conducive to ion transport.

High‐resolution transmission electron microscopy (HRTEM) images of the TB‐COF membrane revealed lattice fringes with a spacing of approximately 2.1 nm (Figure 2e,f), consistent with the designed one‐dimensional pore size. The height distribution in the intensity profile further confirms the uniformity of the channel aperture. Electrostatic potential (ESP) analysis, based on electron density, was employed to identify electrophilic and nucleophilic regions (Figure 2g, Figure S3) [51]. The ESP maps show that Bpy‐H^+^ exhibits the most pronounced positive potential (red regions), indicating a highly positive nanochannel environment conducive to Cl^−^ ion adsorption. This characteristic enhances energy conversion efficiency in nanofluidic devices, particularly in acidic conditions. Furthermore, contact angle measurements demonstrated hydrophilic behavior on both sides of the TB‐COF membrane (Figure S4), promoting electrolyte interaction and facilitating rapid ion transport. Numerous studies have shown that due to the hydrophobicity of organic pollutants, hydrophilic groups introduced on membrane surfaces can form hydrogen bonds with water molecules to construct a stable hydration layer at the membrane/liquid interface. This hydration layer can effectively block direct contact between pollutants and the membrane surface, weaken hydrophobic interactions, and thereby reduce the adsorption and deposition of pollutants. Therefore, membranes with strong hydrophilicity usually exhibit better anti fouling ability. Collectively, these results confirm that the nanochannels within the TB‐COF membrane exhibit high ion selectivity and permeability, rendering them highly suitable for osmotic energy harvesting.

### 2.3 Optimization and Optimal Working Status of TB‐COF Membrane

**FIGURE 3:**
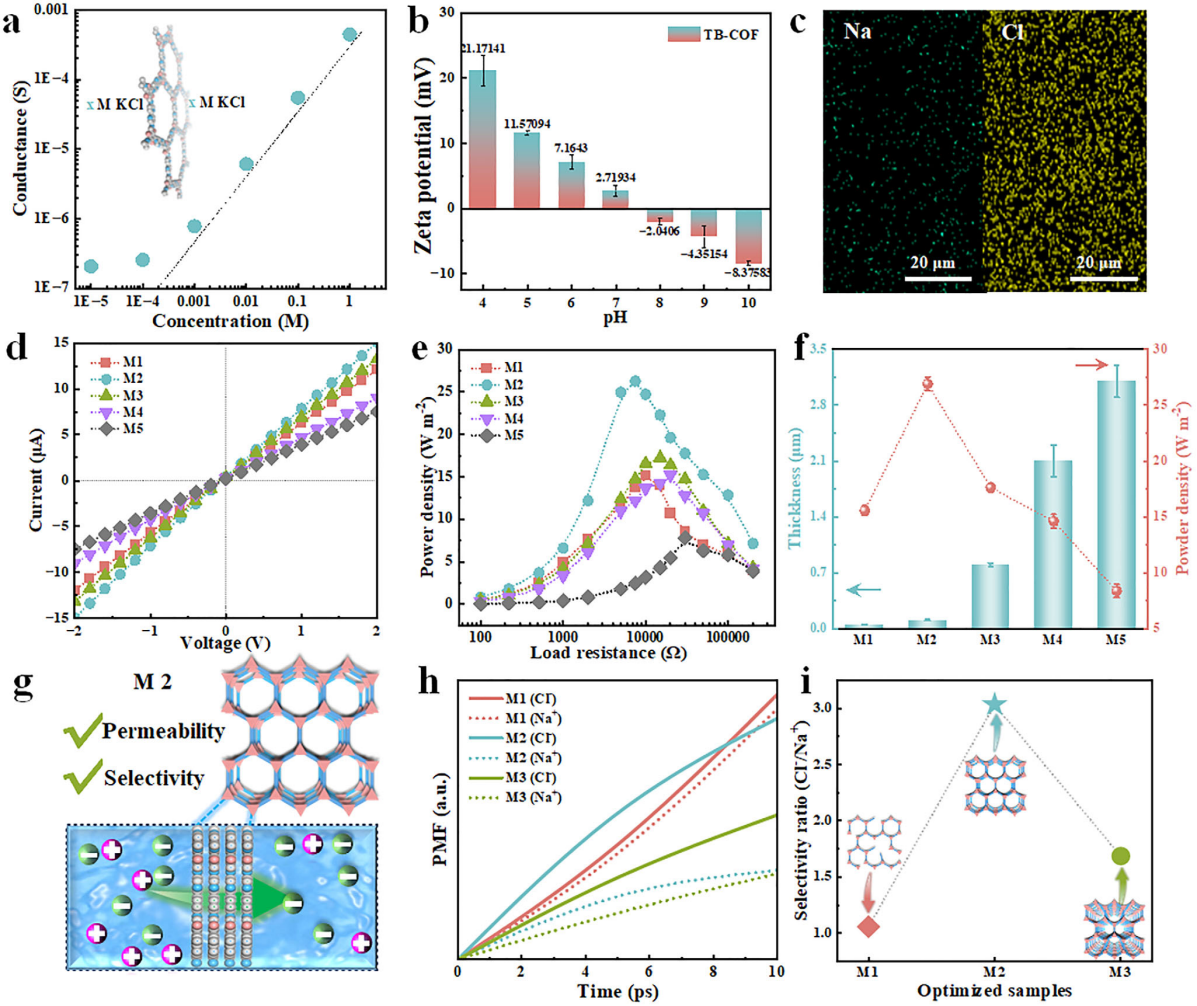
Optimization of TB‐COF membrane and its optimal working state. (a) Ionic conductance of the TB‐COF (M2) membrane under different concentrations of KCl solutions. It deviates away from bulk value at low concentrations, indicating that the ionic transport was controlled by the surface charge. (b) The change in zeta potential of the TB‐COF membrane in relation to pH is consistent with the incorporation of intelligent response groups in the COF. (c) The ion selectivity of the TB‐COF (M2) membrane is demonstrated using two negatively charged NaCl solutions that label the membrane. (d) The *I*–*V* curves (−2 to 2 V) of a series of TB‐COF membranes, prepared by adjusting the concentration of two‐phase monomers in a 0.01 M KCl solution, exhibit ion transport characteristics indicative of the nanochannel. (e) The current density and output power of the M1‐5 membranes vary with load resistance. (f) The power densities of the TB‐COF membranes with varying thicknesses show that the M2 film exhibits exceptional energy harvesting capabilities. (g) MD simulations the behavior of ions passing through the membrane was analyzed in terms of ion permeability and selectivity. The M2 structure maintains an ordered framework and appropriate ion channel length, resulting in excellent ion selectivity and permeability. (h) The PMF profiles illustrate the evolution of the free energy barriers experienced by Cl^−^ and Na^+^ ions as they translocate through the series of TB‐COF membranes over the simulation time. (i) Ion selectivity ratios (Cl^−^/Na^+^) for M1, M2, and M3 based on ion distribution states at 10 ps. (Inset) Schematic illustration of the corresponding TB‐COF membranes, highlighting both the microscopic (internal defects) and macroscopic (thickness) structural features.

As illustrated in Figure 3a, the ion transport behavior of the TB‐COF membrane under varying electrolyte concentrations reveals a strong dependence on surface charge. The uniform nanochannels minimize transport resistance, facilitating efficient ion permeation. In the separation process, not only physical size sieving exists, but also charge mediated electrostatic repulsion becomes one of the key mechanisms for intercepting solutes, improving selectivity and antipollution. To further understand the ion transport properties, we measured the zeta potential of the membrane across a range of pH values (Figure 3b). The membrane exhibits a positive surface charge under neutral conditions, attributable to its intrinsic stacking structure and electron‐inducing effects [52, 53, 54]. Under acidic conditions, protonation of functional groups increases the charge density within the nanochannels, consistent with the observed zeta potential trend. In alkaline environments, hydroxide ions (OH^−^) interact with water to form hydrated clusters that are either free or adsorbed, imparting a slight negative charge to the material [55, 56]. The ion selectivity of the TB‐COF membrane was evaluated as depicted in Figure 3c. The membrane, positioned between NaCl solutions containing oppositely charged ions, was subjected to elemental imaging after rinsing. Remove the membrane from the solution and rinse the surface with deionized water to remove any physically adsorbed salts. EDS can detect ions firmly bound in the nanochannels inside the membrane, rather than ions physically adsorbed on the surface. The detected Cl element signal mainly comes from the strong electrostatic interaction with positively charged pores, which captures Cl^−^ ions inside the pores. However, due to being repelled by positively charged membranes, Na^+^ has a very low concentration in the pores, resulting in a weak signal. Results indicate preferential retention of anions (Cl^−^) and exclusion of cations (Na^+^), confirming charge‐selective transport. To optimize membrane performance, we fabricated TB‐COF membranes with controlled thicknesses by varying monomer concentrations (denoted M1–M5; thicknesses: 55.6 nm, 114.0 nm, 805.7 nm, 2.1 µm, and 3.1 µm; see Table S1, Figure S5). Except for M1, which exhibited structural instability due to insufficient monomer concentration during interfacial polymerization, all membranes were dense, continuous, and crack‐free, as confirmed by SEM (Figure S6). Atomic force microscopy (AFM) further revealed a smooth surface with a roughness of approximately 5–10 nm (Figure S7). As illustrated in the figure. AFM The surface of the display film is smooth (with a roughness of 5–10 nm), which is not conducive to the adhesion of pollutants. To better evaluate the ion transport properties of the aforementioned series of membrane structures, ion transport properties were characterized via current–voltage (*I*–*V)* measurements in 0.01 M KCl under applied biases from −2 to 2 V (Figure 3d). The M2 membrane demonstrated optimal ion transport performance among the series. The symmetric *I*–*V* curves indicate bidirectional and consistent ion transport behavior across the membrane.

To elucidate the charge transport behavior of the TB‐COF membranes, we performed electrochemical measurements at scan rates ranging from 20 to 600 mV s^−1^. The linear relationship between peak current and the square root of the scan rate (Figure S8) indicates diffusion‐controlled ion transport. Thinner membranes reduce ion transport distance and resistance, leading to enhanced transmembrane ion flux [57, 58]. The variation in current density (Figure S9) and output power (Figure 3e) of M1‐5 with load resistance (R_L_) at a salinity gradient of 50‐fold (0.01 and 0.5 M NaCl) is crucial for further investigating the permeability energy conversion performance and optimizing membrane structure. The current density decreases with the increase in the external resistance. The output power density (P) was calculated by P = I^2^R_L_A^−1^, where A is the membrane cross‐sectional area. According to Ohm's law, maximum power output (P_max_) occurs when the external resistance matches the internal membrane resistance [59, 60]. The power density, charge density, and resistance exhibited by M2 are superior to those of other films, with the output power density reaching a P_max_ of 28.85 W m^−^^2^ at a resistance of approximately 10 kΩ. The output power density initially increased with decreasing thickness, peaking for M2, which displayed a defect‐free and continuous structure (Figure 3f). Further increase in membrane thickness raised internal resistance and reduced ion permeability, deteriorating device performance. Additionally, under larger salinity gradients, both current and power density improved significantly, with M2 consistently outperforming M4 (Figure S10).

To elucidate the ion transport mechanism, we performed molecular dynamics (MD) simulations to study NaCl permeation through TB‐COF membranes during structural optimization [61]. Analysis of the trajectories revealed distinct ion selectivity and permeability across the nanochannels (Figure 3g, Figure S11). The M1‐3 layer is sandwiched between NaCl aqueous solution and deionized water. Starting from random configuration, the system stops after running for 10 ps (Figures S12 and S13). The potential of mean force (PMF) profiles for Na^+^ and Cl^−^ ions translocating through the series of TB‐COF membranes, evaluated over the same simulation time, reveal the energy barriers and wells encountered during transmembrane migration (Figure 3h). Meanwhile, to better illustrate ion permeability and selectivity, the Cl^−^/Na^+^ permeability ratios for M1–M3 were calculated at 10 ps. M2 exhibited the highest ratio of 3.03, significantly outperforming M1 (1.06) and M3 (1.69) (Figure 3i). This result correlates with structural characteristics: M1, synthesized at critically low monomer concentration, exhibits structural defects that compromise ion selectivity. In contrast, M2 achieves an optimal balance between permeability and selectivity, while the increased thickness of M3 elevates ion transport resistance and reduces permeability [54, 62].

### 2.4 Transmembrane Ion Transport Behavior and Efficient Osmotic Energy Conversion in Multi‐response Environment

**FIGURE 4:**
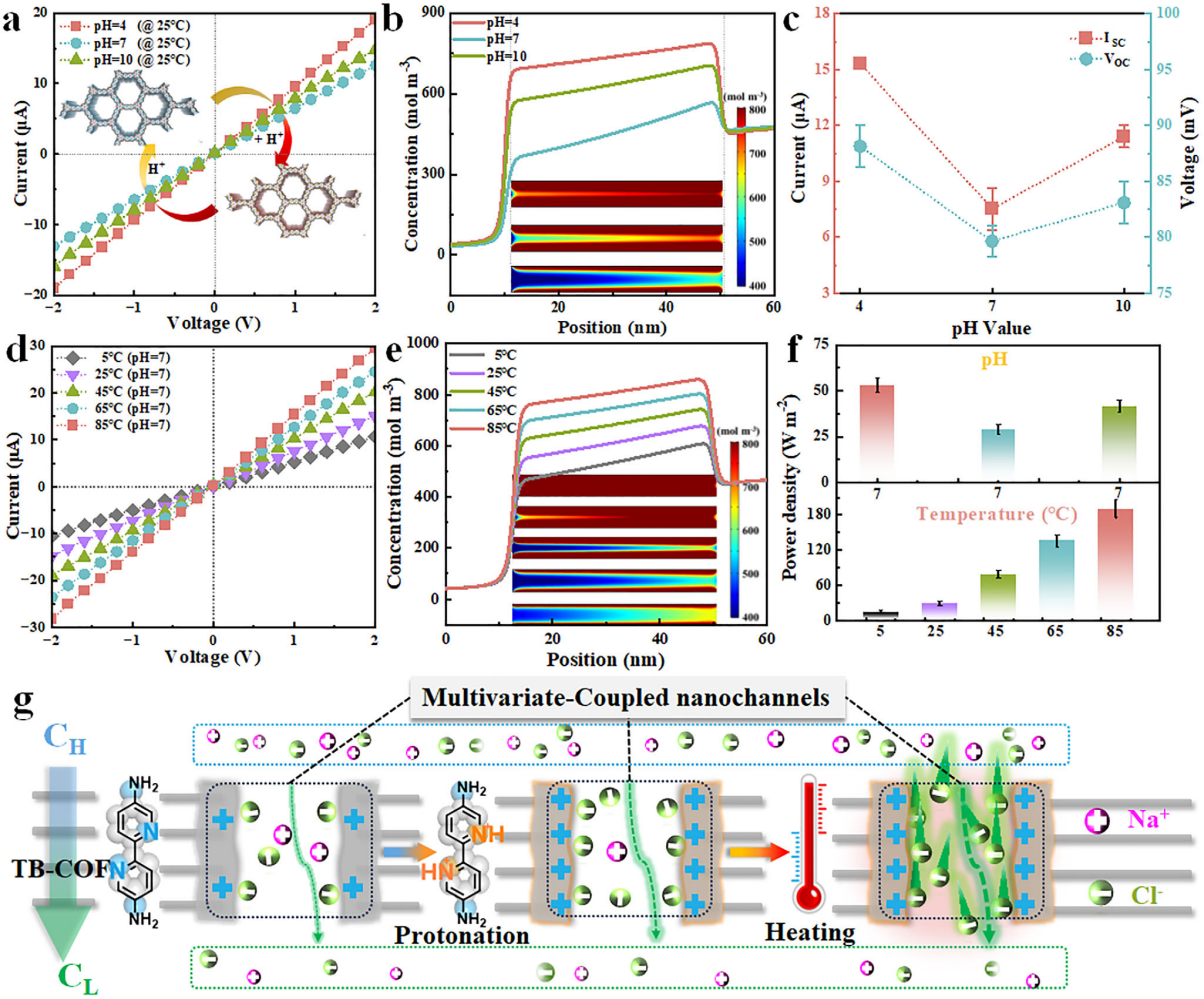
Ionic nanogate property of the TB‐COF membranes in response to the environment. (a) *I–V* curves of M2 in a 0.01 M KCl solution at pH levels of 4, 7, and 10, with a sweep voltage ranging from −2 to 2 V. (b) The simulation results for charge density and pore size within the nanochannel as a function of pH indicate that, under identical pore size conditions, a high charge density enhances ionic current density. The inset illustrates the simulated ion distribution within nano ion channels across different pH environments. (c) Open circuit battery voltage and short‐circuit current under varying pH conditions. (d) *I–V* curves of M2 in a 0.01 M KCl solution at temperatures of 5°C−85°C, with a sweep voltage ranging from −2 to 2 V. Under extreme temperature conditions, the linear variation curve confirms that the working membrane maintains a uniform nanochannel. (e) The simulation results for charge density and pore size within the nanochannel, as temperature varies, indicate that, under identical pore size conditions, a high charge density contributes to improved ionic current density. The inset demonstrates the simulated ion distribution within nano ion channels at different temperature environments. (f) The output power density of the TB‐COF membrane under salinity gradients of river water (0.01 M NaCl) and seawater (0.5 M NaCl) exhibits excellent osmotic energy harvesting performance under conditions of pH 4 and 85°C. (g) The changes in functional groups within TB‐COF and their behavior during ion transport in response to various environmental changes.

The functional groups in TB‐COF enable tunable charge density in response to pH [12, 63]. When tested using 0.01 M KCl solutions at pH 4, 7, and 10, the M2 membrane maintained structural integrity and exhibited stable current output under a fixed voltage of 2 V (Figure S14). *I–V* curves collected from −2 to 2 V in 0.2 V steps demonstrated highly linear and enhanced ion transport performance under acidic conditions (pH  4; Figure 4a, Figure S15), attributable to protonation of functional groups that increases nanochannel charge density. Numerical simulations under symmetric 0.01 M KCl conditions further elucidated the ion transport behavior across pH values (Figure S16a–c, Scheme S2 Supporting Information). Both experimental and simulated results (Figure 4b, Figure S16d–f) showed the highest ion current at pH 4, consistent with increased positive surface charge promoting anion adsorption. It can also be observed that as the charge density increases, the adsorbed ion concentration also increases, and an asymmetric charge distribution appears in the pores. The above confirms that charge density is the core element determining the interaction between ions and the inner wall of the pore. Under alkaline conditions, hydrated OH^−^ clusters contributed to cation adsorption, leading to moderate ion currents. These trends align with zeta potential measurements. Open‐circuit voltage (*V_OC_*) and short‐circuit current (*I_SC_*) were also pH‐dependent (Figure 4c). The highest values (*V_OC_* = 88.15 mV, *I_SC_* = 15.33 µA) were achieved at pH 4, where enhanced protonation elevates pore charge density, thereby improving ion selectivity and permeability. Additionally, scan rate‐dependent tests of M2 in 0.01 M KCl at pH 4–10 and voltages from 110 to 150 mV revealed stable and reproducible electrochemical responses (Figures S17 and S18), confirming robust membrane operation under varying pH and enhanced charge density under acidic conditions. The D was highest at pH 4, indicating that Cl^−^ diffuses more rapidly under acidic conditions. This is consistent with the protonation of bipyridine groups in the COF channels at low pH, which increases the positive surface charge density and enhances anion‑selective transport.

To improve the utilization and recovery of energy from low‐grade heat, we optimized osmotic energy harvesting by regulating the ambient temperature [15, 64, 65]. The *I–V* characteristics of M2 in 0.01 M KCl remained linear across temperatures (Figure 4d), indicating structural stability and sustained ion transport performance even at 85°C. Numerical simulations corroborated these experimental results (Figure S19a–c). Further simulations of surface charge distribution as a function of temperature and pore size revealed that at 85°C and fixed pore diameter, ion current increased significantly, accompanied by enhanced anion accumulation along the pore surface (Figure 4e, inset; Figure S19d–f). This supports our understanding of ion wall interactions promoted at high temperatures. This is mainly attributed to the fact that an increase in temperature significantly reduces the viscosity of the electrolyte solution, thereby reducing the fluid dynamic resistance of ions. This increases ion mobility, making it easier for ions to approach and interact with charged channel walls. Meanwhile. An increase in temperature will partially destroy the hydration shell around the ions, reducing their effective hydration radius. A smaller effective radius allows ions to approach the charged hole wall more closely, thereby enhancing the electrostatic interactions at each encounter [14]. The stable performance of TB‐COF membranes under gradual heating was further confirmed. M4 exhibited consistent increases in current density and maximum power output under a 50‐fold salinity gradient with rising temperature (Figure S20), demonstrating the generalizability of thermally enhanced ion transport. CV of M2 from 5 to 85°C in 0.01 M KCl (110–150 mV) showed stable response across scan rates (Figures S21 and S22), affirming operational reliability under thermal variations. I The derived D values followed the order: 85°C > 65°C > 45°C > 25°C > 5°C. This trend reflects the accelerated ion mobility at elevated temperatures, attributable to reduced solution viscosity and weakened ion‑hydration shells. The result validates that low‑grade thermal energy can effectively boost ion transport and, consequently, osmotic energy conversion [66, 67]. Overall, it is reliable and meaningful for us to carry out the utilization and harvesting of low‐grade heat sources. Finally, TB‐COF membranes were applied for osmotic energy harvesting under a 50‐fold salinity gradient (0.01 M / 0.5 M NaCl, simulating river and seawater conditions) (Figure 4f, Figure S23). Maximum power outputs reached 53.08 W m^−^^2^ at pH 4 and 190.52 W m^−^^2^ at 85°C.

To provide a more comprehensive perspective on the impact of temperature on the conversion and harvesting of osmotic energy. We harvest osmotic energy in an asymmetric temperature field, as shown in Figure S24. As shown in the Figure S25, Under the largest ΔT of 60°C (25°C | 85°C), the output power density reached 215.00 W m^−2^, representing an 11.39% enhancement compared to the performance under an equivalent symmetric high‐temperature condition. But in order to simplify the thermal control system and fully utilize the entire low‐grade heat environment system of desulfurization waste liquid, the design scheme of this experiment adopts a synchronous heating process on both sides. To demonstrate the comprehensiveness of the experimental design, we have expanded the temperature testing range to 95°C, which is the point close to the boiling point of water, on the basis of the original temperature sequence (5°C, 25°C, 45°C, 65°C, 85°C). This represents the actual upper limit of the water system using low‐grade waste heat and ensures that the equipment is allowed and safe. Measure the current density and output power density values at 95°C under a 50‐fold salinity gradient (0.01 M | 0.5 M NaCl), as shown in Figure S26. The output power density reached 236.27 W m^−2,^ and the current density was 3403 A m^−2^, further setting a new record for permeation energy conversion membranes at low temperatures. In aqueous solutions close to 100°C, the violent evaporation of local water and the generation of bubbles can disrupt the stability of the membrane electrolyte interface. This leads to an increased probability of testing data errors and instrument damage, as shown in Figure S27. In summary, in the current study, we have chosen 85°C as the reliable upper limit for performance reporting, which represents typical industrial wastewater thermal conditions and ensures stable operation of the testing system. As summarized in Figure 4g, the membranes effectively harvest energy from salinity gradients, acidic/alkaline environments, and low‐grade heat via mechanisms including transmembrane ion transport, charge density regulation, and thermally accelerated ion mobility. These results underscore the versatility and high performance of TB‐COF membranes in diverse osmotic energy harvesting scenarios.

### 2.5 Outperformed (Synergistically Enhanced) Energy Harvesting Behavior and Ultra‐Stable Working State

**FIGURE 5:**
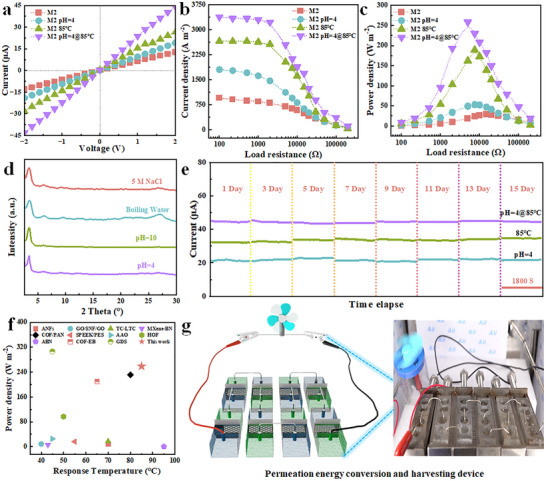
The infiltration energy harvesting device exhibits multivariate‐coupled intelligent responses to changes in pH and temperature, which together improve its long‐term stability and performance. (a) The *I–V* curves of M2 in a 0.01 M KCl solution (at pH 7, pH 4, 85°C, and pH 4 at 85°C) show a voltage sweep from −2 to 2 V. (b and c) The output power and current density of M2 under different external conditions are illustrated. (d) To evaluate the stability of TB COFs, samples were exposed to various chemical environments for 7 days. The positions and intensities of peaks in their XRD spectra remaining unchanged. (e) The current time series data for the energy harvesting device constructed with M2, after being exposed to artificial river water (0.01 M NaCl) and artificial seawater (0.5 M NaCl), indicates that the device maintains a stable output current after operating for 15 days (1800 s per day). (f) A comparison of output power density between the TB‐COF membrane and existing membrane materials under various ambient temperature conditions is presented. (g) By connecting multivariate‐coupled TB‐COF membrane unit cells in series, the device harvests osmotic energy. This energy directly drives a motor propeller, enabling a continuous power supply.

Building on the stable performance of TB‐COF membranes across diverse environments, we designed and simulated a zero‐discharge process for treating desulfurization wastewater from thermal power plants. Based on a multivariate‐coupled design strategy, this approach synergistically utilizes high salinity, acidity, and low‐grade heat inherent in the wastewater for osmotic energy recovery [68, 69]. Ionic transport was evaluated via *I‐V* curves in 0.1 M KCl from −2 to 2 V (Figure 5a). The symmetric current response confirms bidirectional ion permeability of the M2 membrane. Even under aggressive conditions (pH 4, and 85°C), the membrane maintained high ion transport performance. Energy conversion was further assessed under a 50‐fold salinity gradient (0.01 M / 0.5 M NaCl) with an external load (R_L_). Current density decreased with increasing R_L_, and the output power density reached a maximum (P_max_) at the matched resistance, consistent with Ohm's law. Notably, M2 achieved a current density of 3380.43 A m^−^^2^ and a P_max_ of 258.81 W m^−^^2^ under the combined pH 4 and 85°C condition (Figure 5b,c). This high‐performance stems from the multivariate‐coupled design, enabling waste energy reuse and offering a viable solution to alleviate energy demands. XRD confirmed the membrane's crystallinity remains intact after exposure to high‐concentration salt (5 M NaCl), boiling water, acidic/alkaline solutions, and waste heat (Figure 5d), attributable to the robust β‐ketoenamine linkages. Continuous operation over 15 days under various conditions yielded stable current output (Figure 5e), demonstrating long‐term reliability. To better simulate real desulfurization wastewater, we introduced a gradient concentration of SO_4_^2^^−^ ions into our system and compared the ion transport performance and permeation energy conversion efficiency of the TB‐COF membrane (Figures S28 and S29). We found that the presence of sulfate ions increased transport resistance to some extent. Additionally, we prepared a mixed electrolyte solution containing NaCl, Na_2_SO_4_, MgCl_2_, and MgSO_4_ to observe the effects of various salt ions on the nanofluidic channels. Membrane regeneration was achieved through low‐voltage operation (Figure S30), which is crucial for mitigating ion deposition and pore blockage, thereby maintaining long‐term operational stability.

As shown in Figure 5f and Table S2, the TB‐COF membrane achieves a maximum output power density of 258.81 W m^−^^2^ under optimized conditions, surpassing all previously reported osmotic energy conversion materials across a wide range of temperatures [2, 3, 7, 10, 14, 15, 65, 70, 71, 72, 73]. This exceptional performance is attributed to the well‐ordered nanochannels of the two‐dimensional COF structure, which facilitate efficient ion diffusion. The β‐ketoenamine‐linked framework, functionalized with responsive groups along the channel walls, leverages low‐grade thermal energy to simultaneously enhance ion selectivity and permeability, enabling highly efficient energy conversion. To demonstrate practical applicability, we constructed a simulated osmotic energy harvesting device by connecting multiple TB‐COF membrane units in series (Figure 5g) [21, 60, 74, 75, 76]. Each unit was placed between artificial river water (0.01 M NaCl) and seawater (0.5 M NaCl). The integrated device successfully powered a motor propeller, requiring a starting voltage of 0.5 V, and operated continuously with stable output, highlighting its potential for real‐world energy recovery applications.

## 3 Conclusion

In summary, we demonstrate a multivariate coupling design strategy to fabricate TB‐COF membranes with tunable charge density and robust adaptability for harvesting osmotic energy from waste sources. Notably, the Bpy functional groups, uniformly distributed within the TB‐COF nanochannels, provide abundant sites for charge regulation, thereby strongly enhancing transmembrane ion transport. This results in a power output of 53.08 W m^−^^2^ at pH 4. Furthermore, the β‐ketoenamine linkage endows the COF structure with exceptional stability to withstand environmental perturbations, ensuring stable operation and a power density of 190.52 W m^−^^2^ under low‐grade heat. Simulating the multi‐physical field coupling (waste heat and acidic environment) of real industrial waste liquors, the TB‐COF membrane achieved a maximum power output of 258.81 W m^−^^2^. This work illustrates the construction of membranes with superior selectivity and permeability through interfacial assembly. By implementing a policy‐oriented multivariate coupling design, we have developed a nanofluidic device with ultrahigh ionic flux for energy conversion, opening a new avenue for the design of adaptive crystalline porous materials.

## 4 Experimental Section

### 4.1 Materials

All chemicals and reagents were commercially purchased from suppliers and used without further purification. 2,4,6‐Triformylphloroglucinol (Tp), 2,2’‐Bipyridine‐5,5’‐diamine (Bpy), and p‐Toluenesulfonic acid monohydrate (PTSA) was purchased from Energy Chemical. Dichloromethane (DCM, CH_2_Cl_2_), Acetonitrile (ACN), Acetic Acid (HAc), N, N‐Dimethylacetamide (DMAC), Methanol (MeOH) and Acetone (CH_3_COCH_3_) were purchased XiLong Scientific. High purity water (H_2_O) with a resistivity of 18.2 MΩ cm^−1^ was obtained from the Milli‐Q purification system (Millipore, Billerica, MA, USA).

### 4.2 Fabrication of the TB‐COF Membranes

A typical interfacial polymerization reaction process is as follows. Tp (4.20 mg, 0.02 mmol) was dissolved in CH_2_Cl_2_ (100 mL) and placed at the bottom of a 300 mL beaker. Bpy (5.60 mg, 0.03 mmol) and PTSA (11.40 mg, 0.06 mmol) were dissolved in CAN/H_2_O (30 mL / 70 mL), and HAc (20 µL) was added at the same time. The above two‐phase solutions were stirred for 30 min, and the interface reaction system was placed in a beaker (300 mL). CAN/H_2_O (18 mL / 42 mL) was added as a buffer layer between the two‐phase interfaces to reduce the interface reaction rate and increase the crystallinity of TB‐COF Membranes. After the addition of the above components, the reaction was carried out at room temperature for 96 h. After the reaction, the two‐phase solution was removed, and DMAC, CAN, MeOH, and CH_3_COCH_3_ were used for solvent replacement in sequence; each solvent was replaced three times, soaking for 12 h each time. To better remove unreacted monomers, small aggregates and particles. Finally, the TB‐COF membrane (M2) is transferred to the substrate for use. The preferred process for the preparation of TB‐COF membranes (M1, M3, M4, and M5) is in the Supporting Information Table S1.

### 4.3 Characterization

The crystal structures of the samples were studied using X‐ray diffraction (XRD, Empyrean, PANalytical B.V., Germany). Molecular structures and types of functional groups were analyzed using Fourier transform infrared spectroscopy (FTIR, iS10, Thermo Fisher Scientific, USA). The successful preparation of the framework structure can be confirmed by ^13^C solid‐state NMR spectrometer (^13^C SSNMR, Bruker‐AVANCE III HD 600 MHz, Germany). The diffraction lattice fringes were clearly observed using a transmission electron microscope (TEM, JEM 2100 F, JEOL Ltd., Japan). The surface areas and pore sizes of the samples were evaluated using N_2_ adsorption/desorption isotherms at 77 K (BET, ASAP 2460, Micromeritics, USA), after degassing them at 120°C for 24 h under vacuum. Working film were observed using scanning electron microscopy (SEM, Nova Nano 450, FEI, USA), and energy dispersive X‐ray (EDX) analysis was conducted to analyze the surface elemental composition. Atomic force microscopy provides a clearer picture of film thickness and surface roughness (AFM, BRUKER Icon‐XR, USA). Zeta potential surface measuring instrument (Zeta, Sur PASS 3 Eco, Anton Paar Company, Austria) measured the surface Zeta potential of the membrane as a function of pH. The thermal stability was characterized using the DSC Q2000 (TA Instruments, LLC, USA) and the PerkinElmer Pyris 1 TGA apparatus (Waltham, Massachusetts, USA). The contact angle meter (DSA 25S Krüss GmbH) measures the water contact angle of membrane samples and analyzes the wettability of the material. Cyclic voltammetry (CV, Electrochemical workstation CHI660E, CHN) better observes the transport process of ions under different environmental and parameter conditions.

### 4.4 Electrical Measurements

The ionic current through the membrane was measured by a Keithley 6487 picoammeter (Keithley Instruments, Cleveland, OH). The membrane was fixed in the connector between two compartments as a separator. Electrolyte solutions were prepared with deionized water (18.2 MΩ∙cm^−1^, MilliQ). The transmembrane potential was provided by a pair of Ag | AgCl electrodes with equal electrolytes placed on the two sides of the membrane. Sweeping voltages ranging from −2 to 2 V were applied across the membrane. When the electrolytic cells on both sides have the same concentration of KCl electrolyte, the ion transport properties across the membrane can be measured, and when a linear *I–V* curve appears, it indicates that the membrane has a symmetrical structure. Additionally, both sides of the membrane were placed between unequal electrolytes, namely artificial seawater (0.5 M NaCl) and artificial river water (0.01 M NaCl) to simulate the confluence of rivers and oceans for building energy harvesting generators. The collected electrical energy is then transferred to an external circuit. And provide electronic load. It is known that the maximum power that can be extracted occurs when the ionic internal resistance of the membrane is equal to the external load resistance (*R*). The power density calculation formula of the resistor in the circuit is *P = I^2^R*.

### 4.5 Osmotic Energy Conversion and Harvesting under Various Salinity Gradient Conditions

Evaluate the output performance of the TB‐COF membrane for energy harvesting through permeation by connecting it to an external resistor. The formula for calculating the power density of circuit resistors is *P*_output_ = *I* ^2^ × *R*_L_. A 0.01 M NaCl solution was added to one side of the electrolytic cell as a low‐concentration electrolyte (simulating artificial river water), while 0.05 M NaCl, 0.5 M NaCl, and 5 M NaCl solutions were added to the other side as high‐concentration electrolytes, creating a series of salinity gradients (5‐fold, 50‐fold, and 500‐fold). The resulting permeability performance at different concentrations was evaluated. Due to the significant driving force, as the concentration gradient increases, both the current density and power density rise, demonstrating the variation trend of composite nanofluid devices across the series of salinity gradients.

## Conflicts of Interest

The authors declare no conflicts of interest.

## Supporting information




**Supporting File 1**: advs73733‐sup‐0001‐SuppMat.docx.

**Supporting File 2**: advs73733‐sup‐0002‐VideoS1.mp4.

## Data Availability

The data that support the findings of this study are available in Supporting Information of this article.

## References

[1] X. Lu , Z. Zhang , T. Hiraki , et al., “A Solid‐State Electrolysis Process for Upcycling Aluminium Scrap,” Nature 606 (2022): 511–515, 10.1038/s41586-022-04748-4. 35417651

[2] B. Yu , J. Duan , H. Cong , et al., “Thermosensitive Crystallization–Boosted Liquid Thermocells for Low‐Grade Heat Harvesting,” Science 370 (2020): 342–346, 10.1126/science.abd6749. 32913001

[3] C. Chen , G. Yang , D. Liu , et al., “Aramid Nanofiber Membranes for Energy Harvesting From Proton Gradients,” Advanced Functional Materials 32 (2021): 202102080.

[4] K. Wang , H. Yang , Z. Liao , et al., “Monolayer‐Assisted Surface‐Initiated Schiff‐Base‐Mediated Aldol Polycondensation for the Synthesis of Crystalline Sp2 Carbon‐Conjugated Covalent Organic Framework Thin Films,” Journal of the American Chemical Society 145 (2023): 5203–5210, 10.1021/jacs.2c12186. 36779889

[5] R. Xing , X. Zhang , X. Fan , R. Xie , L. Wu , and X. Fang , “Coupling Strategies of Multi‐Physical Fields in 2D Materials‐Based Photodetectors,” Advanced Materials 37 (2025): 2501833, 10.1002/adma.202501833. 40059460

[6] H. Ling , W. Xin , X.‐Y. Kong , L. Jiang , and L. Wen , “Engineering Biomimetic Nanofluidics Toward High‐Performance Osmotic Energy Harvesting,” Advanced Materials 37 (2025): 2506029, 10.1002/adma.202506029. 40589385

[7] X. Zuo , C. Zhu , W. Xian , et al., “Thermo‐Osmotic Energy Conversion Enabled by Covalent‐Organic‐Framework Membranes With Record Output Power Density,” Angewandte Chemie International Edition 61 (2022): 2116910, 10.1002/anie.202116910. 35179288

[8] S. Kandambeth , A. Mallick , B. Lukose , M. V. Mane , T. Heine , and R. Banerjee , “Construction of Crystalline 2D Covalent Organic Frameworks With Remarkable Chemical (Acid/Base) Stability via a Combined Reversible and Irreversible Route,” Journal of the American Chemical Society 134 (2012): 19524–19527, 10.1021/ja308278w. 23153356

[9] X. Zhu , J. Zhong , J. Hao , et al., “Polymeric Nano‐Blue‐Energy Generator Based on Anion‐Selective Ionomers With 3D Pores and pH‐Driving Gating,” Advanced Energy Materials 10 (2020): 2001552, 10.1002/aenm.202001552.

[10] C. Chen , D. Liu , L. He , et al., “Bio‐Inspired Nanocomposite Membranes for Osmotic Energy Harvesting,” Joule 4 (2020): 247–261, 10.1016/j.joule.2019.11.010.

[11] C. Zhang , H.‐Q. Liang , Z.‐K. Xu , and Z. Wang , “Harnessing Solar‐Driven Photothermal Effect Toward the Water–Energy Nexus,” Advanced Science 6 (2019): 1900883, 10.1002/advs.201900883. 31572646 PMC6760470

[12] X. Zhu , Y. Zhou , J. Hao , et al., “A Charge‐Density‐Tunable Three/Two‐Dimensional Polymer/Graphene Oxide Heterogeneous Nanoporous Membrane for Ion Transport,” ACS Nano 11 (2017): 10816–10824, 10.1021/acsnano.7b03576. 29039923

[13] L. Ding , D. Xiao , Z. Zhao , Y. Wei , J. Xue , and H. Wang , “Ultrathin and Ultrastrong Kevlar Aramid Nanofiber Membranes for Highly Stable Osmotic Energy Conversion,” Advanced Science 9 (2022): 2202869, 10.1002/advs.202202869. 35780505 PMC9443462

[14] H. J. Wang , Y. Zhang , J. Wang , et al., “In Situ Synthesized HOF Ion Rectification Membrane with Ultrahigh Permselectivity for Nanofluidic Osmotic Energy Harvesting,” Advanced Functional Materials 35 (2024): 2412477.

[15] W. Xin , H. Xiao , X.‐Y. Kong , et al., “Biomimetic Nacre‐Like Silk‐Crosslinked Membranes for Osmotic Energy Harvesting,” ACS Nano 14 (2020): 9701–9710, 10.1021/acsnano.0c01309. 32687698

[16] L. Cao , H. Wu , Y. Cao , et al., “Weakly Humidity‐Dependent Proton‐Conducting COF Membranes,” Advanced Materials 32 (2020): 2005565, 10.1002/adma.202005565. 33179394

[17] T. Y. Liu and G. L. Liu , “Porous Organic Materials Offer Vast Future Opportunities,” Nature Communications 11 (2020): 4984, 10.1038/s41467-020-15911-8. PMC7532140 33009391

[18] H. Qi , J. Zhong , W. Sun , et al., “Integrated Device for Osmotic Energy Collection and Detection Based on the Metal–Organic Framework of Nanoconfinement Channels,” CCS Chemistry 7 (2025): 1424, 10.31635/ccschem.024.202404323.

[19] B. Dong , Y. Pei , N. Mansour , et al., “Deciphering Nanoconfinement Effects on Molecular Orientation and Reaction Intermediate by Single Molecule Imaging,” Nature Communications 10 (2019): 4815, 10.1038/s41467-019-12799-x. PMC6811571 31645571

[20] M. Hao , X. G. Xiong , Z. Li , et al., “Adsorption‐Catalysis Synergy Boosting the Conversion of Polysulfide over Mesoporous Carbon Confined Molecular Catalysts,” Advanced Energy Materials 15 (2025): 2501226.

[21] X. Zhu , J. Hao , B. Bao , et al., “Unique Ion Rectification in Hypersaline Environment: A High‐Performance and Sustainable Power Generator System,” Science Advances 4 (2018): aau1665, 10.1126/sciadv.aau1665. PMC6203222 30397649

[22] J. Yang , B. Tu , G. Zhang , et al., “Advancing Osmotic Power Generation by Covalent Organic Framework Monolayer,” Nature Nanotechnology 17 (2022): 622–628, 10.1038/s41565-022-01110-7. 35469012

[23] J. Xia , H. Gao , S. Pan , et al., “Light‐Augmented Multi‐Ion Interaction in Mxene Membrane for Simultaneous Water Treatment and Osmotic Power Generation,” ACS Nano 17 (2023): 25269–25278, 10.1021/acsnano.3c08487. 38071658

[24] Z. Zhang , L. P. Wen , and L. Jiang , “Nanofluidics for Osmotic Energy Conversion,” Nature Reviews Materials 6 (2021): 622–639, 10.1038/s41578-021-00300-4.

[25] Y. Zhou and L. Jiang , “Bioinspired Nanoporous Membrane for Salinity Gradient Energy Harvesting,” Joule 4 (2020): 2244–2248, 10.1016/j.joule.2020.09.009.

[26] J. Zhong , H. Qi , T. Xu , et al., “Space Charge Improved Poly(Aryl Ether Sulfone) Composite Membrane for Osmotic Energy Conversion,” Chinese Journal of Chemistry 43 (2025): 814–822, 10.1002/cjoc.202401024.

[27] P. Wang , K. Zhang , J. Liao , et al., “Mesoscale Dynamics of Electrosorbed Ions in Fast‐Charging Carbon‐Based Nanoporous Electrodes,” Nature Nanotechnology 20 (2025): 1228–1236, 10.1038/s41565-025-01947-8. 40550973

[28] Z. Zhou , T. Ma , H. Zhang , et al., “Carbon Dioxide Capture From Open Air Using Covalent Organic Frameworks,” Nature 635 (2024): 96–101, 10.1038/s41586-024-08080-x. 39443804

[29] Y. P. Ying , S. B. Peh , H. Yang , et al., “Ultrathin Covalent Organic Framework Membranes via a Multi‐Interfacial Engineering Strategy for Gas Separation,” Advanced Materials 34 (2021): 202104946. 10.1002/adma.202104946 34535914

[30] Y. Zhang , H. Wang , W. Wang , et al., “Engineering Covalent Organic Framework Membranes for Efficient Ionic/Molecular Separations,” Matter 7 (2024): 1406.

[31] W.‐R. Cui , C.‐R. Zhang , W. Jiang , et al., “Regenerable and Stable Sp2 Carbon‐Conjugated Covalent Organic Frameworks for Selective Detection and Extraction of Uranium,” Nature Communications 11 (2020): 436, 10.1038/s41467-020-14289-x. PMC6978342 31974343

[32] C. Q. Ji , C. J. Kang , B. C. Patra , et al., “Flexible Covalent Organic Frameworks: Design, Synthesis, and Applications,” CCS Chemistry 6 (2024): 856.

[33] W. Liu , X. Li , P. He , et al., “Synthesis of Carboxyl‐Functionalized COFs With Alternate Stable β‐Ketoenamine and Benzimidazole Linkages: Unraveling Exceptional Solvent Effects for Efficient Uranium Separation,” Small 20 (2024): 2403684, 10.1002/smll.202403684. 39096108

[34] T. H. Huang , J. F. Kou , H. Yuan , et al., “Linker Modulation of Covalent Organic Frameworks at Atomic Level for Enhanced and Selective Photocatalytic Oxidation of Thioether,” Advanced Functional Materials (2024): 2413943.

[35] J. Á. Martín‐Illán , L. Sierra , A. Guillem‐Navajas , et al., “β‐Ketoenamine‐Linked Covalent Organic Frameworks Synthesized via Gel‐to‐Gel Monomer Exchange Reaction: From Aerogel Monoliths to Electrodes for Supercapacitors,” Advanced Functional Materials 34 (2024): 2403567, 10.1002/adfm.202403567.

[36] C. R. DeBlase , K. E. Silberstein , T.‐T. Truong , H. D. Abruña , and W. R. Dichtel , “β‐Ketoenamine‐Linked Covalent Organic Frameworks Capable of Pseudocapacitive Energy Storage,” Journal of the American Chemical Society 135 (2013): 16821–16824, 10.1021/ja409421d. 24147596

[37] A. M. Kaczmarek , Y.‐Y. Liu , M. K. Kaczmarek , et al., “Developing Luminescent Ratiometric Thermometers Based on a Covalent Organic Framework (COF),” Angewandte Chemie 132 (2020): 1948–1956, 10.1002/ange.201913983. 31777996

[38] P. Zhang , S. Chen , C. Zhu , et al., “Covalent Organic Framework Nanofluidic Membrane as a Platform for Highly Sensitive Bionic Thermosensation,” Nature Communications 12 (2021): 1844, 10.1038/s41467-021-22141-z. PMC7988099 33758174

[39] S. Hou , W. Ji , J. Chen , Y. Teng , L. Wen , and L. Jiang , “Free‐Standing Covalent Organic Framework Membrane for High‐Efficiency Salinity Gradient Energy Conversion,” Angewandte Chemie International Edition 60 (2021): 9925–9930, 10.1002/anie.202100205. 33527640

[40] R.‐Z. Li , S.‐C. Yu , W. Jiang , et al., “Direct Synthesis of Amide‐Linked COFs via Ester‐Amine Exchange Reaction,” Journal of the American Chemical Society 147 (2025): 31016–31024, 10.1021/jacs.5c08823. 40825095

[41] W. Jiang , J. Zhou , X. Zhong , et al., “Axial Alignment of Covalent Organic Framework Membranes for Giant Osmotic Energy Harvesting,” Nature Sustainability 8 (2025): 446–455, 10.1038/s41893-024-01493-6.

[42] K. Xiao , L. Jiang , and M. Antonietti , “Ion Transport in Nanofluidic Devices for Energy Harvesting,” Joule 3 (2019): 2364.

[43] J. Huang , M. Yuan , Y. Zhang , et al., “Sustainable Nanofiltration Membranes Enable Ultrafast Water Purification,” Nature Water 3 (2025): 1048–1056, 10.1038/s44221-025-00492-x.

[44] L. Xu , Z. Huang , Z. Deng , et al., “A Transparent, Highly Stretchable, Solvent‐Resistant, Recyclable Multifunctional Ionogel With Underwater Self‐Healing and Adhesion for Reliable Strain Sensors,” Advanced Materials 33 (2021): 2105306, 10.1002/adma.202105306. 34647370

[45] C. Zhao , J. Hou , M. Hill , B. Freeman , H. Wang , and H. Zhang , “Enhanced Gating Effects in Responsive Sub‐Nanofluidic Ion Channels,” Accounts of Materials Research 4 (2023): 786–797, 10.1021/accountsmr.3c00067.

[46] J. Lu and H. Wang , “Emerging Porous Framework Material‐Based Nanofluidic Membranes toward Ultimate Ion Separation,” Matter 4 (2021): 2810–2830.

[47] H. Wang , M. Wang , X. Liang , et al., “Organic Molecular Sieve Membranes for Chemical Separations,” Chemical Society Reviews 50 (2021): 5468–5516, 10.1039/D0CS01347A. 33687389

[48] H. Zhang , Z. Lin , P. Kidkhunthod , and J. Guo , “Stable Immobilization of Nickel Ions on Covalent Organic Frameworks for Panchromatic Photocatalytic Hydrogen Evolution,” Angewandte Chemie International Edition 62 (2023): 202217527, 10.1002/anie.202217527. 36960568

[49] J. Yuan , X. You , N. A. Khan , et al., “Photo‐Tailored Heterocrystalline Covalent Organic Framework Membranes for Organics Separation,” Nature Communications 13 (2022): 3826, 10.1038/s41467-022-31361-w. PMC9250524 35780168

[50] K. Dey , M. Pal , K. C. Rout , et al., “Selective Molecular Separation by Interfacially Crystallized Covalent Organic Framework Thin Films,” Journal of the American Chemical Society 139 (2017): 13083–13091, 10.1021/jacs.7b06640. 28876060

[51] Q. Zhang , C.‐Y. Tsai , L.‐J. Li , and D.‐J. Liaw , “Colorless‐to‐Colorful Switching Electrochromic Polyimides With Very High Contrast Ratio,” Nature Communications 10 (2019): 1239, 10.1038/s41467-019-09054-8. PMC6423275 30886136

[52] C. Wang , J. J. Xu , H. Y. Chen , et al., “Mass Transport in Nanofluidic Devices,” Science China Chemistry 55 (2012): 453.

[53] M. Y. Chen , K. Yang , J. Wang , et al., “In Situ Growth of Imine‐Bridged Anion‐Selective COF/AAO Membrane for Ion Current Rectification and Nanofluidic Osmotic Energy Conversion,” Advanced Functional Materials 33 (2023): 2302427.

[54] Q.‐W. Meng , J. Li , Z. Lai , et al., “Optimizing Selectivity via Membrane Molecular Packing Manipulation for Simultaneous Cation and Anion Screening,” Science Advances 10 (2024): ado8658, 10.1126/sciadv.ado8658. PMC11423885 39321297

[55] N. Agmon , H. J. Bakker , R. K. Campen , et al., “Protons and Hydroxide Ions in Aqueous Systems,” Chemical Reviews 116 (2016): 7642. 27314430 10.1021/acs.chemrev.5b00736 PMC7116074

[56] Y. Xia , D. Zhu , C. Huang , et al., “Alkaline‐Resistant Covalent Organic Framework Membranes for Selective Hydroxide Ion Separation,” Chemical Engineering Journal 498 (2024): 155458, 10.1016/j.cej.2024.155458.

[57] S. Gong , H. Liu , F. Zhao , et al., “Vertically Aligned Bismuthene Nanosheets on Mxene for High‐Performance Capacitive Deionization,” ACS Nano 17 (2023): 4843–4853, 10.1021/acsnano.2c11430. 36867670

[58] Z. Zhang , W. Shen , L. Lin , et al., “Vertically Transported Graphene Oxide for High‐Performance Osmotic Energy Conversion,” Advanced Science 7 (2020): 2000286. 32596122 10.1002/advs.202000286 PMC7312320

[59] S. Lin , Z. Wang , L. Wang , et al., “Salinity Gradient Energy Is Not a Competitive Source of Renewable Energy,” Joule 8 (2024): 334.

[60] J. Zhong , T. Xu , H. Qi , et al., “Permeability and Selectivity Synergistically Enhanced Nanofluidic Membrane for Osmotic Energy Harvesting,” Carbon Energy 6 (2024): 458, 10.1002/cey2.458.

[61] J. Lin , W. Ye , S. Xie , et al., “Shielding Effect Enables Fast Ion Transfer Through Nanoporous Membrane for Highly Energy‐Efficient Electrodialysis,” Nature Water 1 (2023): 725–735, 10.1038/s44221-023-00113-5.

[62] X. Xu , Y. Chen , Z. Wang , et al., “Nanofiltration Membranes With Ultra‐High Negative Charge Density for Enhanced Anion Sieving and Removal of Organic Micropollutants,” Nature Water 3 (2025): 704–713, 10.1038/s44221-025-00440-9.

[63] Y. Zhang , P. He , M. Zhang , et al., “Mild and Subtle Synthesis of β‐Ketoenamine COFs With High Crystallinity and Controllable Solubility Guided by a Monomer Preassembly Strategy,” Small 20 (2024): 2407874, 10.1002/smll.202407874. 39428841

[64] R. Hu , D. Xu , and X. Luo , “Liquid Thermocells Enable Low‐Grade Heat Harvesting,” Matter 3 (2020): 1400.

[65] W. Xian , X. Zuo , C. Zhu , et al., “Anomalous Thermo‐Osmotic Conversion Performance of Ionic Covalent‐Organic‐Framework Membranes in Response to Charge Variations,” Nature Communications 13 (2022): 3386, 10.1038/s41467-022-31183-w. PMC9192728 35697704

[66] S. Yin , J. Li , Z. Lai , et al., “Giant Gateable Thermoelectric Conversion by Tuning the Ion Linkage Interactions in Covalent Organic Framework Membranes,” Nature Communications 15 (2024): 8137, 10.1038/s41467-024-52487-z. PMC11408633 39289381

[67] T. Li , X. Zhang , S. D. Lacey , et al., “Cellulose Ionic Conductors With High Differential Thermal Voltage for Low‐Grade Heat Harvesting,” Nature Materials 18 (2019): 608–613, 10.1038/s41563-019-0315-6. 30911121

[68] L. Chai , Z. Li , K. Wang , et al., “Ultra‐Fast Recyclable and Value‐Added Desulfation Method for Spent Lead Paste via Dual Intensification Processes,” Advanced Science 10 (2023): 2304863, 10.1002/advs.202304863. 37867231 PMC10700223

[69] J. Feng , J. Yang , S. Cui , et al., “Waste to Wealth: Efficient Wet Desulfurization of Electric Arc Furnace Dust (Eafd) and Recycling of Desulfurization Solid Residue,” Chemical Engineering Journal 491 (2024): 152053, 10.1016/j.cej.2024.152053.

[70] G. Yang , D. Liu , C. Chen , et al., “Stable Ti3C2Tx Mxene–Boron Nitride Membranes With Low Internal Resistance for Enhanced Salinity Gradient Energy Harvesting,” ACS Nano 15 (2021): 6594–6603, 10.1021/acsnano.0c09845. 33787220

[71] Y. Sun , Y. Wu , Y. Hu , et al., “Thermoenhanced Osmotic Power Generator via Lithium Bromide and Asymmetric Sulfonated Poly(Ether Ether Ketone)/Poly(Ether Sulfone) Nanofluidic Membrane,” NPG Asia Materials 13 (2021): 50, 10.1038/s41427-021-00317-9.

[72] N. Van Toan , M. M. I. M. Hasnan , D. Udagawa , et al., “Thermoelectric Power Battery Using Al2O3 Nanochannels of 10 Nm Diameter for Energy Harvesting of Low‐Grade Waste Heat,” Energy Conversion and Management 199 (2019): 111979, 10.1016/j.enconman.2019.111979.

[73] X. Yang , J. Song , Y. Liu , et al., “Molecularly Engineered Rigid Ultra‐Micropore Membranes for Ultrahigh‐Power Osmotic Energy Harvesting From High‐Temperature Hypersaline Brine,” Advanced Materials 37 (2025): 2505485, 10.1002/adma.202505485. 40343408

[74] F. Zhang , J. Yu , Y. Si , et al., “Meta‐Aerogel Ion Motor for Nanofluid Osmotic Energy Harvesting,” Advanced Materials 35 (2023): 2302511. 10.1002/adma.202302511 37295070

[75] N. Sheng , M. Zhang , Q. Song , et al., “Enhanced Salinity Gradient Energy Harvesting With Oppositely Charged Bacterial Cellulose‐Based Composite Membranes,” Nano Energy 101 (2022): 107548, 10.1016/j.nanoen.2022.107548.

[76] W.‐X. Pan , L. Chen , W.‐Y. Li , et al., “Scalable Fabrication of Ionic‐Conductive Covalent Organic Framework Fibers for Capturing of Sustainable Osmotic Energy,” Advanced Materials 36 (2024): 2401772, 10.1002/adma.202401772. 38634168

