# Supplementary material for: Ionic Highways under Multivariate‐Coupled Strategies: Ultrahigh Power Generation from Industrial Waste Liquors Using Robust COF Membranes
Source: Adv Sci (Weinh). 2026 Jan 9;13(16):e23044. doi: 10.1002/advs.202523044 (PMC13042868; doi:10.1002/advs.202523044)
Supplement: Supplementary file 1 — Supporting File 1: advs73733‐sup‐0001‐SuppMat.docx. [file ADVS-13-e23044-s002.docx]

Supporting Information

**Ionic highways under Multivariate-Coupled Strategies: Ultrahigh Power Generation from Industrial Waste Liquors using Robust COF Membranes**

*Hongyan Qi, Weibo Sun, Jundong Zhong, Zhen Xing, Tingting Xu, Shuang Zhao, Haoyang Tan, Ziyi Hu, Hujun Qian, Yuchen Wu, Haibo Zhang, Jianxin Mu, Xuanbo Zhu*, and Lei Jiang*

**Supporting Information contains:**

Supporting Section

Supporting Schemes S1 - S2

Supporting Figures S1 - S30

Supporting Tables S1 - S2

Supporting Video S1

**Supporting Section**

**Materials**

All chemicals and reagents were commercially purchased from suppliers and used without further purification. 2,4,6-Triformylphloroglucinol (Tp), 2,2’-Bipyridine-5,5’-diamine (Bpy), and p-Toluenesulfonic acid monohydrate (PTSA) was purchased from Energy Chemical. Dichloromethane (DCM, CH_2_Cl_2_), Acetonitrile (ACN), Acetic Acid (HAc), N, N-Dimethylacetamide (DMAC), Methanol (MeOH) and Acetone (CH_3_COCH_3_) were purchased XiLong Scientific. High purity water (H_2_O) with a resistivity of 18.2 MΩ cm^-1^ was obtained from the Milli-Q purification system (Millipore, Billerica, MA, USA).

**Fabrication of the TB-COF Membranes**

A typical interfacial polymerization reaction process is as follows. Tp (4.20 mg, 0.02 mmol) was dissolved in CH_2_Cl_2_ (100 mL) and placed at the bottom of a 300 mL beaker. Bpy (5.60 mg, 0.03 mmol) and PTSA (11.40 mg, 0.06 mmol) were dissolved in CAN/H_2_O (30 mL / 70 mL), and HAc (20 μL) was added at the same time. The above two-phase solutions were stirred for 30 min, and the interface reaction system was placed in a beaker (300 mL). CAN / H_2_O (18 mL / 42 mL) was added as a buffer layer between the two-phase interfaces to reduce the interface reaction rate and increase the crystallinity of TB-COF Membranes. After the addition of the above components, the reaction was carried out at room temperature for 96 h. After the reaction, the two-phase solution was removed, and DMAC, CAN, MeOH, and CH_3_COCH_3_ were used for solvent replacement in sequence; each solvent was replaced three times, soaking for 12 h each time. To better remove unreacted monomers, small aggregates and particles. Finally, the TB-COF membrane (M2) is transferred to the substrate for use. The preferred process for the preparation of TB-COF membranes (M1, M3, M4, and M5) is in the Supporting Information **Table S1**.

**Characterization**

The crystal structures of the samples were studied using X-ray diffraction (XRD, Empyrean, PANalytical B.V., Germany). Molecular structures and types of functional groups were analyzed using Fourier transform infrared spectroscopy (FTIR, iS10, Thermo Fisher Scientific, USA). The successful preparation of the framework structure can be confirmed by ^13^C solid-state NMR spectrometer (^13^C SSNMR, Bruker-AVANCE III HD 600 MHz, Germany). The diffraction lattice fringes were clearly observed using a transmission electron microscope (TEM, JEM 2100 F, JEOL Ltd., Japan). The surface areas and pore sizes of the samples were evaluated using N_2_ adsorption/desorption isotherms at 77 K (BET, ASAP 2460, Micromeritics, USA), after degassing them at 120°C for 24 h under vacuum. Working film were observed using scanning electron microscopy (SEM, Nova Nano 450, FEI, USA), and energy dispersive X-ray (EDX) analysis was conducted to analyze the surface elemental composition. Atomic force microscopy provides a clearer picture of film thickness and surface roughness (AFM, BRUKER Icon-XR, USA). Zeta potential surface measuring instrument (Zeta, Sur PASS™ 3 Eco, Anton Paar Company, Austria) measured the surface Zeta potential of the membrane as a function of pH. The thermal stability was characterized using the DSC Q2000 (TA Instruments, LLC, USA) and the PerkinElmer Pyris 1 TGA apparatus (Waltham, Massachusetts, USA). The contact angle meter (DSA 25S Krüss GmbH) measures the water contact angle of membrane samples and analyzes the wettability of the material. Cyclic voltammetry (CV, Electrochemical workstation CHI660E, CHN) better observes the transport process of ions under different environmental and parameter conditions.

**Electrical Measurements**

The ionic current through the membrane was measured by a Keithley 6487 picoammeter (Keithley Instruments, Cleveland, OH). The membrane was fixed in the connector between two compartments as a separator. Electrolyte solutions were prepared with deionized water (18.2 MΩ∙cm^-1^, MilliQ). The transmembrane potential was provided by a pair of Ag | AgCl electrodes with equal electrolytes placed on the two sides of the membrane. Sweeping voltages ranging from -2 V to 2 V were applied across the membrane. When the electrolytic cells on both sides have the same concentration of KCl electrolyte, the ion transport properties across the membrane can be measured, and when a linear *I-V* curve appears, it indicates that the membrane has a symmetrical structure. Additionally, both sides of the membrane were placed between unequal electrolytes, namely artificial seawater (0.5 M NaCl) and artificial river water (0.01 M NaCl) to simulate the confluence of rivers and oceans for building energy harvesting generators. The collected electrical energy is then transferred to an external circuit. And provide electronic load. It is known that the maximum power that can be extracted occurs when the ionic internal resistance of the membrane is equal to the external load resistance (*R*). The power density calculation formula of the resistor in the circuit is *P=I^2^R*.

**Permeation Energy Conversion and Harvesting Under Various Salinity Gradient Conditions**

Evaluate the output performance of the TB-COF membrane for energy harvesting through permeation by connecting it to an external resistor. The formula for calculating the power density of circuit resistors is P_output_ = I^2^ × R_L_. A 0.01 M NaCl solution was added to one side of the electrolytic cell as a low-concentration electrolyte (simulating artificial river water), while 0.05 M NaCl, 0.5 M NaCl, and 5 M NaCl solutions were added to the other side as high-concentration electrolytes, creating a series of salinity gradients (5-fold, 50-fold, and 500-fold). The resulting permeability performance at different concentrations was evaluated. Due to the significant driving force, as the concentration gradient increases, both the current density and power density rise, demonstrating the variation trend of composite nanofluid devices across the series of salinity gradients.

**Permeation Energy Conversion and Harvesting Based on pH-Responsive Intelligence**

To minimize the influence of hydrogen ion and hydroxide ion concentrations in acid-base solutions on transmembrane ion transport performance, we established pH values lower than those of the salt solution. This approach ensures that salt ions dominate the transport process. The variation in pH is critical to the ion transport mechanism. The functional groups within the framework, along with their unique structures, can modulate changes in charge density during the response process, thereby affecting ion transport to some extent. The conversion and harvesting of osmotic energy in this context align with the workflow described in the section "Conversion and Harvesting of Osmotic Energy under a Series of Salinity Gradients. the electrolyte solution, which includes 0.05 M NaCl, 0.5 M NaCl, and 5 M NaCl, each placed in environments with pH values of 4, 7, and 10.

**Permeation Energy Conversion and Harvesting in Low-Grade Heat Source Environments**

The temperature variations of low-grade heat sources were simulated using a constant temperature metal bath. Low-grade heat sources are typically defined as those with temperatures of ≤ 100°C. In this study, the temperature settings were 5°C, 25°C, 45°C, 65°C, and 85°C for osmotic energy conversion and harvesting. The processes of converting and harvesting osmotic energy align with the workflow described in the section "Conversion and Harvesting of Osmotic Energy under a Series of Salinity Gradient Conditions, with only modifications in the testing environment. As the temperature increases, the viscosity of the liquid decreases, the ion migration rate accelerates, and the ion permeability of the TB-COF membrane increases. Consequently, at a temperature of 85°C, the TB-COF membrane demonstrates exceptional performance in terms of maximum output power density. This response trend is consistent with the multivariate-coupled design strategy.

**Supporting Schemes**

**Electro-chemical Testing Setup**

The equipment and procedures used for both ion‑transport characterization and osmotic energy conversion measurements. A schematic of the overall electrochemical testing setup is provided in Scheme S1.

**
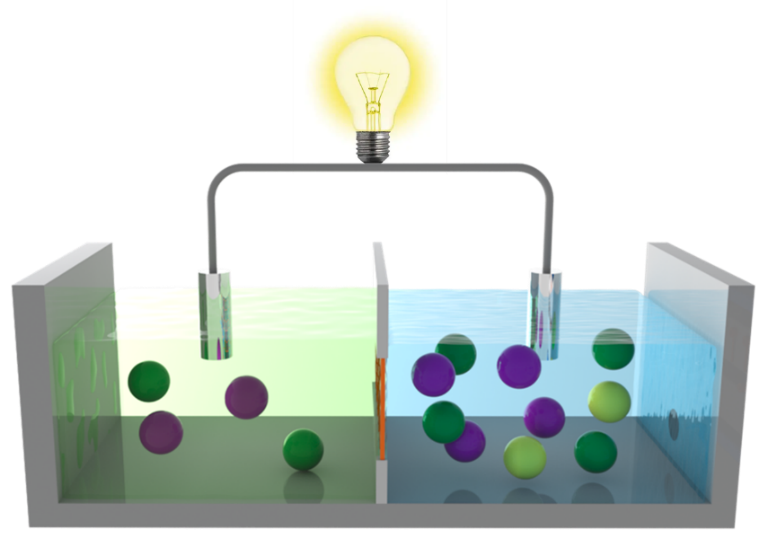
**

**Scheme S1.** Schematic of the electro-chemical testing setup.

**1. Ion‑Transport Characterization Setup**

**Membrane Cell:** The TB‑COF membrane was mounted as a separator in a custom‑made connector between two identical electrolyte compartments (volume: ~3 mL each).

**Electrolytes:** All solutions were prepared using deionized water (18.2 MΩ·cm^-1^, Milli‑Q).

**Current Measurement:** The ionic current through the membrane was recorded using a Keithley 6487 picoammeter (Keithley Instruments, Cleveland, OH).

**Electrodes:** A pair of Ag | AgCl electrodes was placed on each side of the membrane to apply the transmembrane potential.

**Voltage Sweep:** A linear voltage sweeps from –2 V to +2 V was applied, and the resulting current was measured. A linear I‑V response under symmetric electrolyte conditions (e.g., 0.01 M KCl on both sides) confirmed the symmetric structure of the membrane.

Testing Conditions for Multi‑Physical Fields:

**Salinity Gradient:** Symmetric solutions (0.01 M KCl on both sides) were used to study surface‑charge‑dependent transport; concentration series from 10⁻⁵ M to 1 M KCl were also employed.

**pH Gradient:** The same electrolyte (0.01 M KCl) was adjusted to pH 4, 7, and 10 using HCl or KOH, and placed in both compartments.

**Temperature Gradient:** The cell was placed in a temperature‑controlled chamber. Measurements were taken at set temperatures of 278.15 K, 298.15 K, 318.15 K, 338.15 K, and 350.15 K, with 0.01 M KCl in both compartments.

**2. Osmotic Energy Conversion (Power Generation) Setup**

**Cell Assembly:** The TB‑COF membrane served as the separator between two chambers containing electrolytes with different concentrations (e.g., 0.01 M | 0.5 M NaCl).

**Electrodes & Circuit:** Ag | AgCl electrodes were connected to an external circuit. The open‑circuit voltage (*V_OC_*) and short‑circuit current (*I_SC_*) were measured using the same Keithley 6487 picoammeter.

**Power Measurement:** An external load resistor (R) was applied to the circuit. The output power (P) was calculated as P=I^2^R. The maximum extractable power was determined when the external load resistance matched the internal resistance of the membrane system.

Multi‑Field Energy Harvesting Conditions:

**Salinity Gradient:** Concentration ratios of 5‑fold, 50‑fold, and 500‑fold were tested (0.01 M | 0.05 M, 0.01 M | 0.5 M, and 0.01 M | 5 M NaCl).

pH Gradient: A fixed salinity gradient (0.01 M | 0.5 M NaCl) was used while adjusting the pH of both solutions to 4, 7, or 10.

**Temperature Gradient:** The same salinity gradient (0.01 M | 0.5 M NaCl) was maintained, and the whole cell was placed in a temperature‑controlled environment with the same set of temperatures as above (5℃ - 5℃).

**Numerical Simulations**

The numerical simulation experiment was carried out “Electrostatics (AC / DC Module)” and “Nernst-Planck without Electroneutrality” modules. To simplify the calculated system, an assuming steady-state condition was implemented. Ion transport in the nanopore is governed by the concentration difference and surface charge, while the ion transport through the nanopore can be dominated by the Poisson and Nernst-Planck (PNP) equations at equilibrium. PNP equations are shown below:

$$\begin{aligned} \boldsymbol{\nabla}^{\boldsymbol{2}}\boldsymbol{\phi=}\boldsymbol{-}\frac{\boldsymbol{\rho}_{\boldsymbol{\nu}}}{\boldsymbol{\varepsilon}_{\boldsymbol{0}}\boldsymbol{\varepsilon}_{\boldsymbol{r}}}\boldsymbol{\#}\left( \boldsymbol{1} \right) \end{aligned}$$

$$\begin{aligned} \boldsymbol{J}_{\boldsymbol{i}}\boldsymbol{=}\boldsymbol{-}\boldsymbol{D}_{\boldsymbol{i}}\left( \boldsymbol{\nabla}\boldsymbol{c}_{\boldsymbol{i}}\boldsymbol{+}\frac{\boldsymbol{Z}_{\boldsymbol{i}}\boldsymbol{F}\boldsymbol{c}_{\boldsymbol{i}}}{\boldsymbol{k}_{\boldsymbol{B}}\boldsymbol{T}}\boldsymbol{\nabla}\boldsymbol{\phi} \right)\boldsymbol{\#}\left( \boldsymbol{2} \right) \end{aligned}$$

Where *ɛ_0_*, *ɛ_r_*, *F*, *k_B_*, *T* are the vacuum permittivity, the relative permittivity of water, Faraday constant, Boltzmann constant, temperature. And *φ*, *ρ_ν_*, *J_i_*, *c_i_*, *D_i_*, *Z_i_* are the electrical potential, space charge density, ionic flux, ion concentration, diffusion coefficient and valence of ionic species i, respectively.

The system work in a steady state, then

$$\begin{aligned} \boldsymbol{\nabla}\boldsymbol{J}_{\boldsymbol{i}}\boldsymbol{=0}\boldsymbol{\#}\left( \boldsymbol{3} \right) \end{aligned}$$

The sum of transmembrane ionic flux perpendicular to the inner nanopore can be calculated

$$\begin{aligned} \boldsymbol{J}_{\boldsymbol{i}}\boldsymbol{=}\int_{\boldsymbol{R}} \boldsymbol{d}\boldsymbol{J}_{\boldsymbol{i}}\boldsymbol{\#}\left( \boldsymbol{4} \right) \end{aligned}$$

According to the previous work, for that 2D model, the units of the right part of the equation ($\sum_{i} FZ_{i}\vec{J}_{i}$) is A m^-1^, thus, the calculated value of the left part is the cationic or anionic current density perpendicular to the entrance of the nanopore. The normal ionic current density perpendicular to the inner of nanopore can be calculated

$$\begin{aligned} \boldsymbol{I}_{\boldsymbol{i}}\boldsymbol{=}\sum_{\boldsymbol{i}} \boldsymbol{F}\boldsymbol{Z}_{\boldsymbol{i}}\boldsymbol{J}_{\boldsymbol{i}} \left( \boldsymbol{i = + or}\boldsymbol{-} \right)\boldsymbol{\#}\left( \boldsymbol{5} \right) \end{aligned}$$

The normal net ionic current density perpendicular to the inner of nanopore can be calculated

$$\begin{aligned} \boldsymbol{I}_{\boldsymbol{net}}\boldsymbol{=}\sum_{\boldsymbol{j}} \boldsymbol{I}_{\boldsymbol{j}} \left( \boldsymbol{j = + and}\boldsymbol{-} \right)\boldsymbol{\#}\left( \boldsymbol{6} \right) \end{aligned}$$

**Simulation Model**

To effectively capture the ion-transport behavior under salinity, pH, and temperature gradients, we adopted a two‑dimensional (2D) nanofluidic channel model. This choice was motivated by the fact that the TB‑COF membrane, prepared via interfacial polymerization, exhibits a highly ordered and layered microstructure with uniformly distributed pores and surface charges. Given the homogeneous laminar flow characteristics observed in such membranes, a 2D laminar‑structure‑based channel model **(Scheme S2)** was constructed to simplify the numerical analysis while retaining the essential physics.


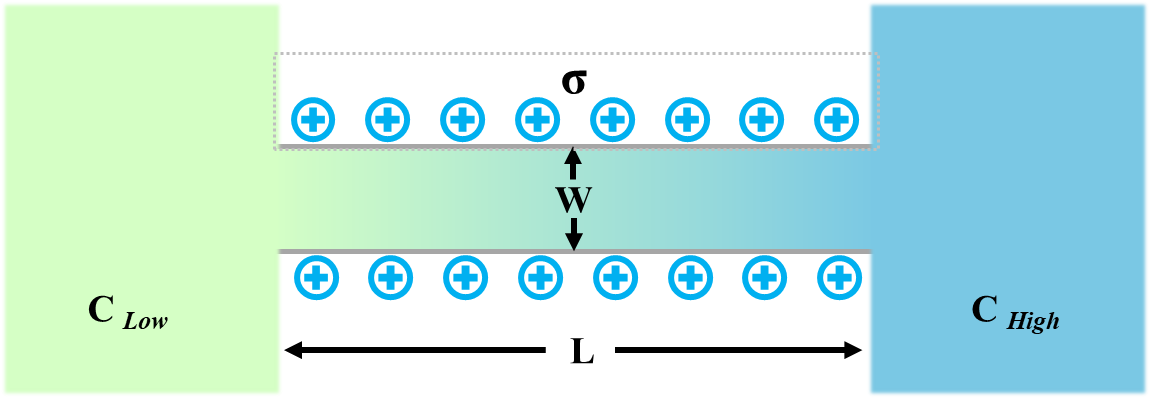


**Scheme S2**. The 2D simulation model based on the PNP theory.

The key components and settings of the model are as follows: Two reservoirs with dimensions of 200 nm x 100 nm are separated by laminar ion channels. The width (M) of the nano ion channel is set to 2.1 nm, and the path through which ions flow is defined by a pore length (L) of 40 nm, ensuring the computational accuracy of the continuum model. During the numerical simulation of acid-base gradient, we set the charge density for pH 4 to 0.46 C m^-2^, pH 7 to 0.06 C m^-2^, and pH 10 to 0.18 C m^-2^ based on the Zeta point test results. The temperature is set to room temperature of 298.15 K, and CHigh and Clow are set to 0.5 M and 0.01 M, respectively. Integrating the above parameters, simulate the process of harvesting osmotic energy. At the same time, in an environment with a temperature gradient, the charge density is set to 0.06 C m^-2^, ensuring that the channel is at a charge density of pH 7. The temperatures were set to 278.15K, 298.15K, 318.15K, 338.15K, and 350.15K, respectively. C_High_ and C_low_ were set to 0.5M and 0.01M, respectively. Based on the above parameter settings, the ability to harvest osmotic energy under a 50-fold salinity gradient was studied. Based on the detailed discussion and presentation of the model for constructing numerical simulations, we believe that your valuable feedback is crucial for improving the quality of the manuscript. This simplified but physically representative model allows us to systematically examine the effects of various external fields (concentration, pH, temperature) on ion distribution, flux, and eventual energy‑conversion performance.

**Supporting Figures**

**Figure S1.** ^13^C solid-state NMR spectra of TB-COF. The spectrum of TB-COF exhibits resonances at 150.6 ppm and 107.4 ppm, corresponding to the enamine carbon and the α-enamine carbon.

**Figure S2.** The thermogravimetric analysis (TGA) spectrum of TB-COF indicates that the collapse temperature of the nanochannel framework is approximately 400°C. The demonstrated excellent thermal stability confirms that independent membrane devices are better suited to meet practical application requirements.


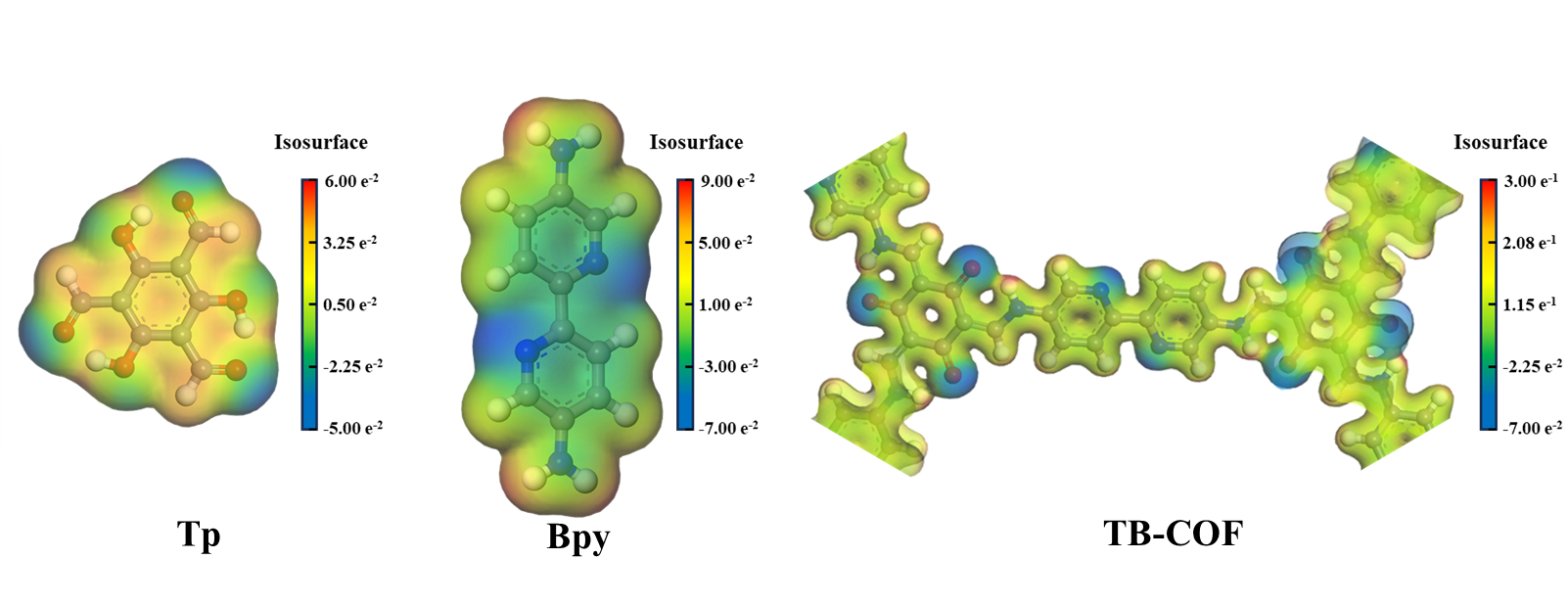


**Figure S3.** The electrostatic potential (ESP) of Tp, Bpy, and TB-COF was calculated using the electron density of the molecules. Typically, the blue shaded areas in the molecule indicate negative electrostatic potential (suggesting locations for electrophilic attack), while the red shaded areas indicate positive electrostatic potential (suggesting locations for nucleophilic attack). The highest and lowest electrostatic potentials of Tp and Bpy are maintained within the smallest unit of TB-COF. The extremely negative potential represents the most favorable position for Cl⁻ adsorption, particularly around the electronegative nitrogen atoms.


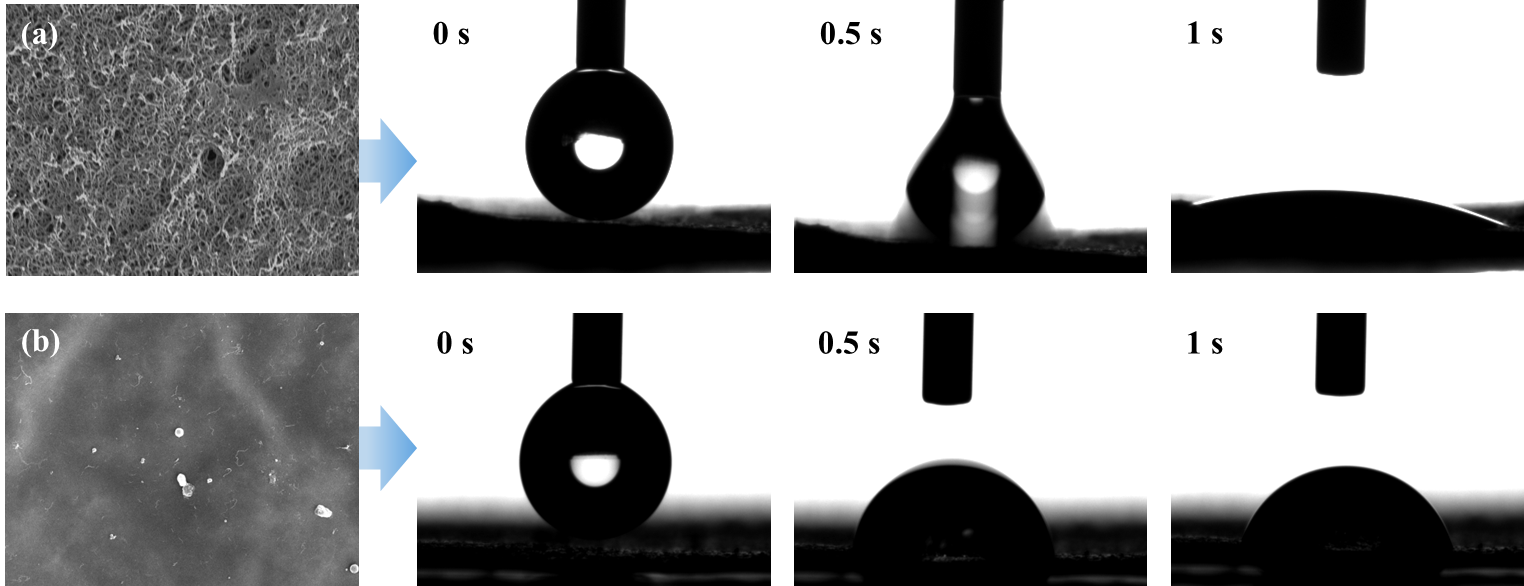


**Figure S4.** The water contact angle on both sides of the TB-COF membrane is illustrated in this figure. **(a)** The bipyridine monomer, a COF monomer, is soluble in water. **(b)** The aldehyde monomer of the COF is soluble in dichloromethane. Since these two synthetic solvents are immiscible, the rate of monomer movement differs when the TB-COF film forms at this interface. Consequently, the macrostructure of the film varies, which is also reflected in their differing hydrophobicity. In the first two seconds, the surface depicted in **Figure (a)** demonstrates significant hydrophilicity.

We characterized the surface properties of TB-COF membrane through water contact angle measurement, and the results are shown in **Figure S4**. The membrane exhibits good hydrophilicity. Numerous studies have shown that due to the hydrophobicity of organic pollutants, hydrophilic groups introduced on membrane surfaces can form hydrogen bonds with water molecules to construct a stable hydration layer at the membrane/liquid interface. This hydration layer can effectively block direct contact between pollutants and the membrane surface, weaken hydrophobic interactions, and thereby reduce the adsorption and deposition of pollutants. Therefore, membranes with strong hydrophilicity usually exhibit better anti fouling ability.


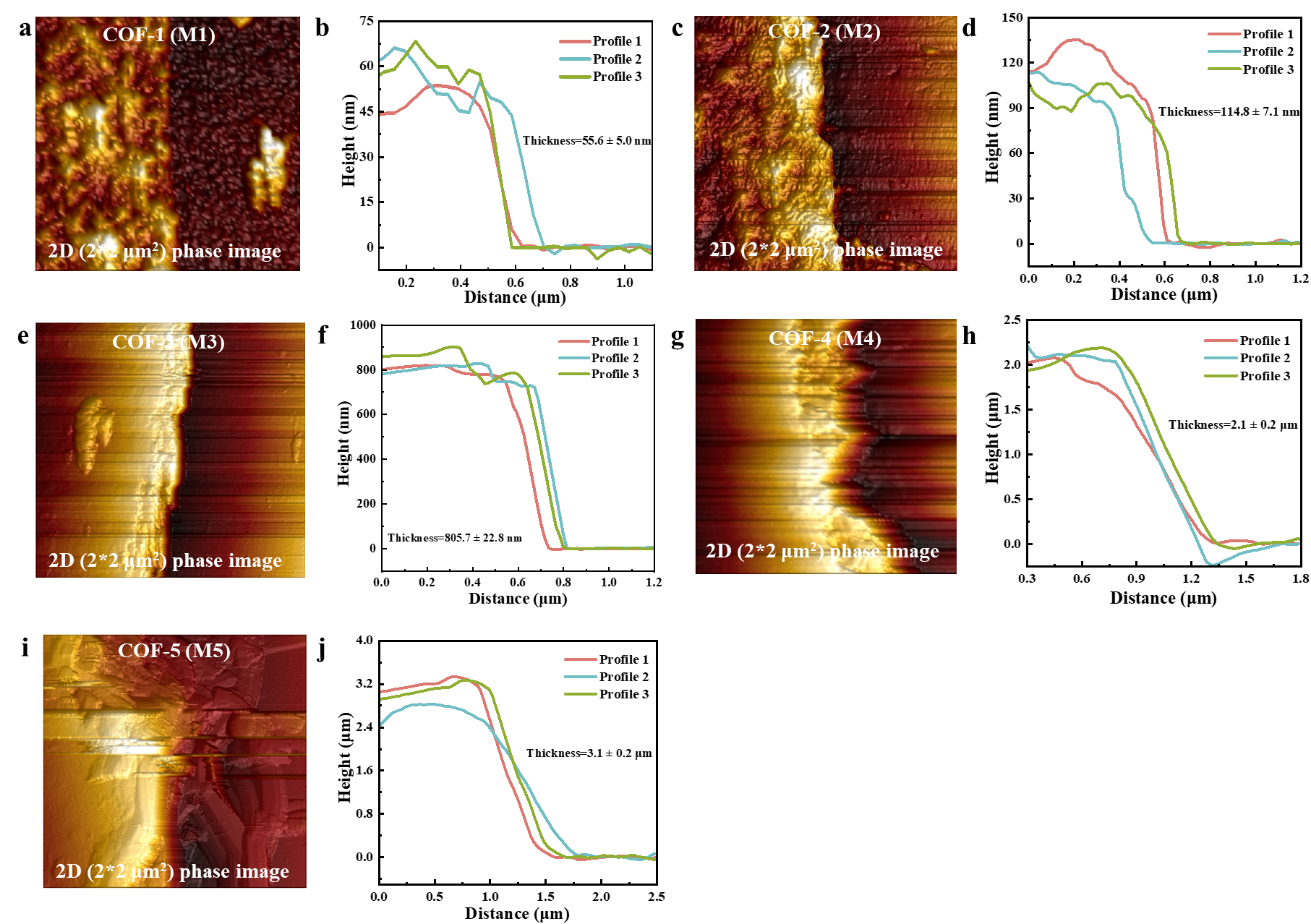


**Figure S5.** The height analysis of the silicon wafer relative to the film surface, conducted using atomic force microscopy, which confirms the film's thickness. Panels **(a)** and **(b)** represent M1 with a thickness of 55.6 ± 5.0 nm; panels **(c)** and **(d)** represent M2 at 114 ± 7.1 nm; panels **(e)** and **(f)** represent M3 at 805.7 ± 22.8 nm; panels **(g)** and **(h)** represent M4 at 2.1 ± 0.2 μm; and panels **(i)** and **(j)** represent M5 at 3.1 ± 0.2 μm. Consequently, by controlling the reaction time and monomer concentration, COF films with thicknesses ranging from tens of nanometers to several micrometers can be readily prepared. Additionally, this analysis indicates that the region between the organic phase and the aqueous phase is within a molecular thickness range during the interfacial reaction process. Therefore, in this confined space, molecular building blocks are compelled to form two-dimensional COF layers with atomic thickness. The layer-by-layer growth of the COF film at the restricted interface results in a self-standing COF film with excellent mechanical robustness.


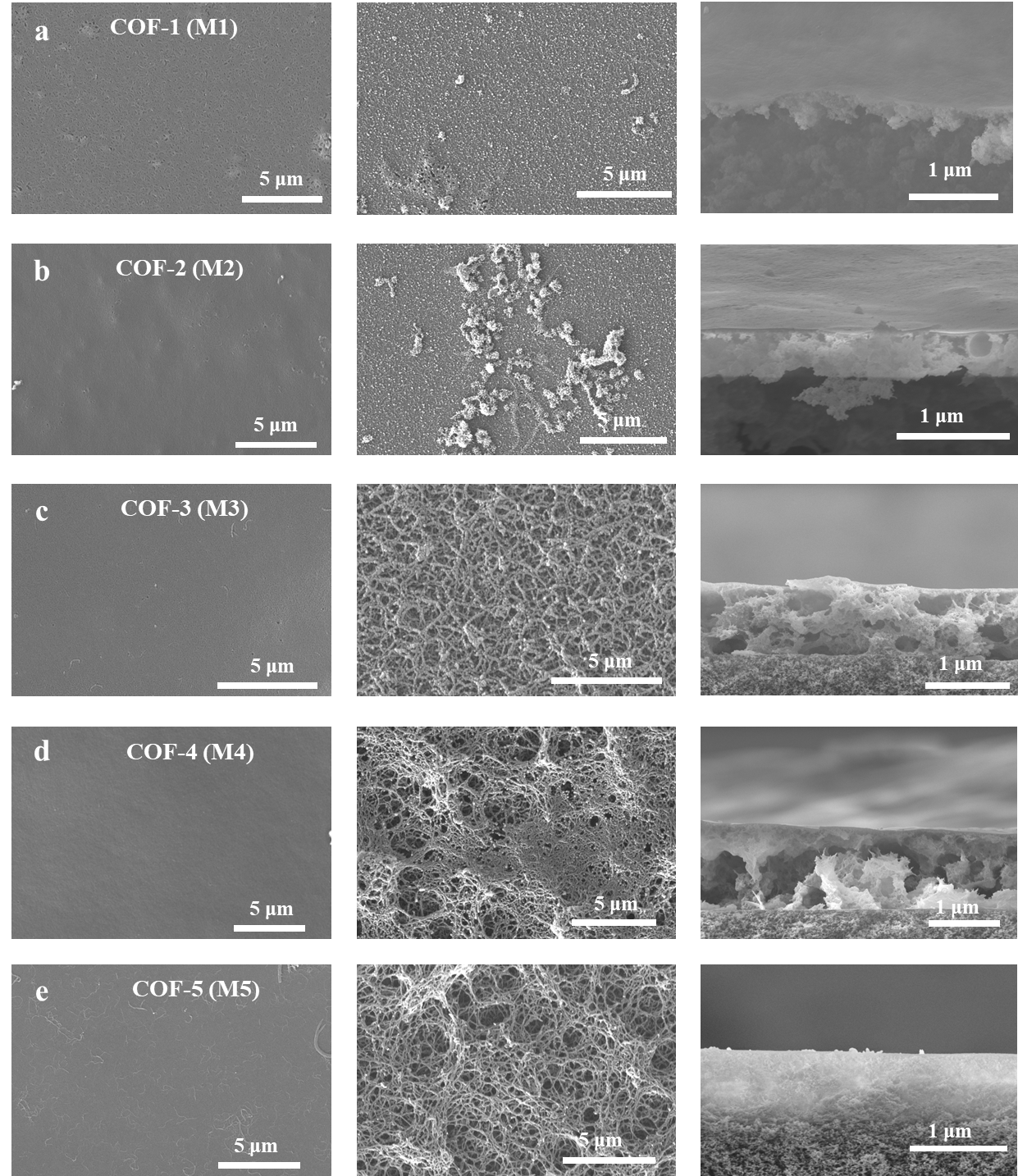


**Figure S6.** To investigate the surface morphology of the COF film in relation to variations in monomer concentration, we examined the surface morphology of both the organic phase and the aqueous phase using scanning electron microscopy. This is illustrated in **Figure (a)**, which represents the M1 organic phase/water phase (and similarly for the others), along with the corresponding cross-sectional view. **Figures. (b-e)** represent M2, M3, M4, and M5, respectively. Comparative observations reveal that M1 exhibits defects and isolated crystal particles on the membrane surface, attributed to the low monomer concentration within the constrained reaction system. As the monomer concentration increases, the defects on the membrane surface (organic phase) diminish, while the presence of impurities increases. Additionally, the rise in polymer-like particles or linear structures on the membrane surface (aqueous phase) contributes to an increase in the mechanical strength of the membrane as its thickness changes.


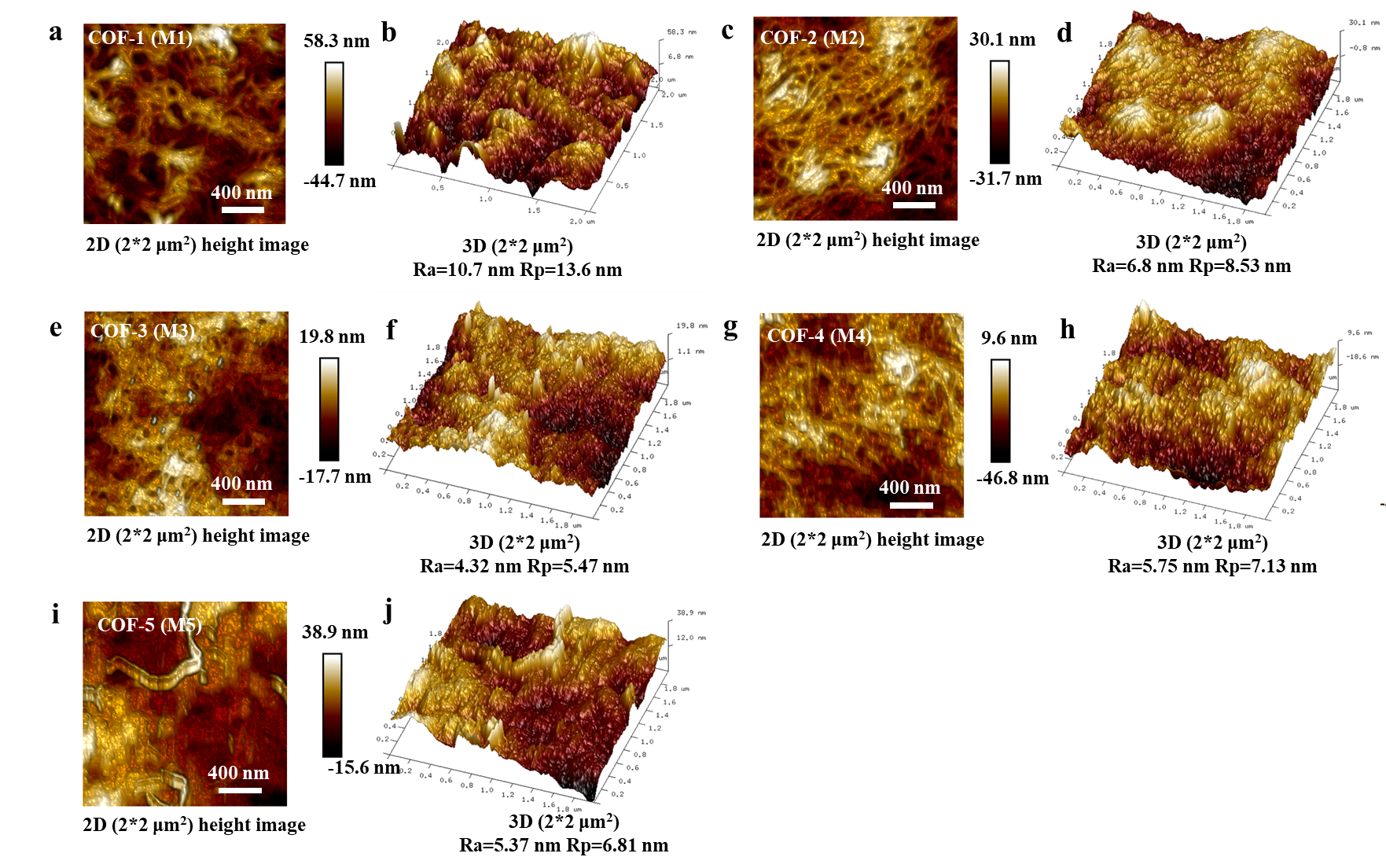


**Figure S7.** To gain a deeper understanding of the internal structure of the film, independent COF thin films were transferred onto a silicon wafer, and their surface morphology was analyzed using atomic force microscopy (AFM), as illustrated in the figure. Panels **(a)** and **(b)** represent M1; **(c)** and **(d)** represent M2; **(e)** and **(f)** represent M3; **(g)** and **(h)** represent M4; **(i)** and **(j)** represent M5. Ra denotes root mean square roughness, while Rp indicates average roughness. Through comparative observation, M1 exhibits high roughness, with its membrane surface displaying defects and isolated crystal particles, consistent with the electron microscopy images. Subsequently, as the membrane undergoes uniform polymerization, its surface roughness decreases and stabilizes, indicating that the surface becomes relatively smooth.

A large number of studies suggest that rough membranes are more susceptible to membrane fouling than photo synovium. On the one hand, the higher roughness of the membrane surface provides a larger surface area for the contact of pollutants; On the other hand, rough surfaces are prone to "dead zones" with low water flow shear stress, making it difficult for pollutants adhered or deposited on the membrane to be washed away, thereby exacerbating irreversible pollution. As shown in **Figure S7**, to gain a deeper understanding of the internal structure of the film, independent COF thin films were transferred onto a silicon wafer, and their surface morphology was analyzed using atomic force microscopy (AFM), as illustrated in the figure. AFM The surface of the display film is smooth (with a roughness of 5-10 nm), which is not conducive to the adhesion of pollutants. Therefore, from the perspective of surface morphology, TB-COF film has a certain antipollution structural foundation.


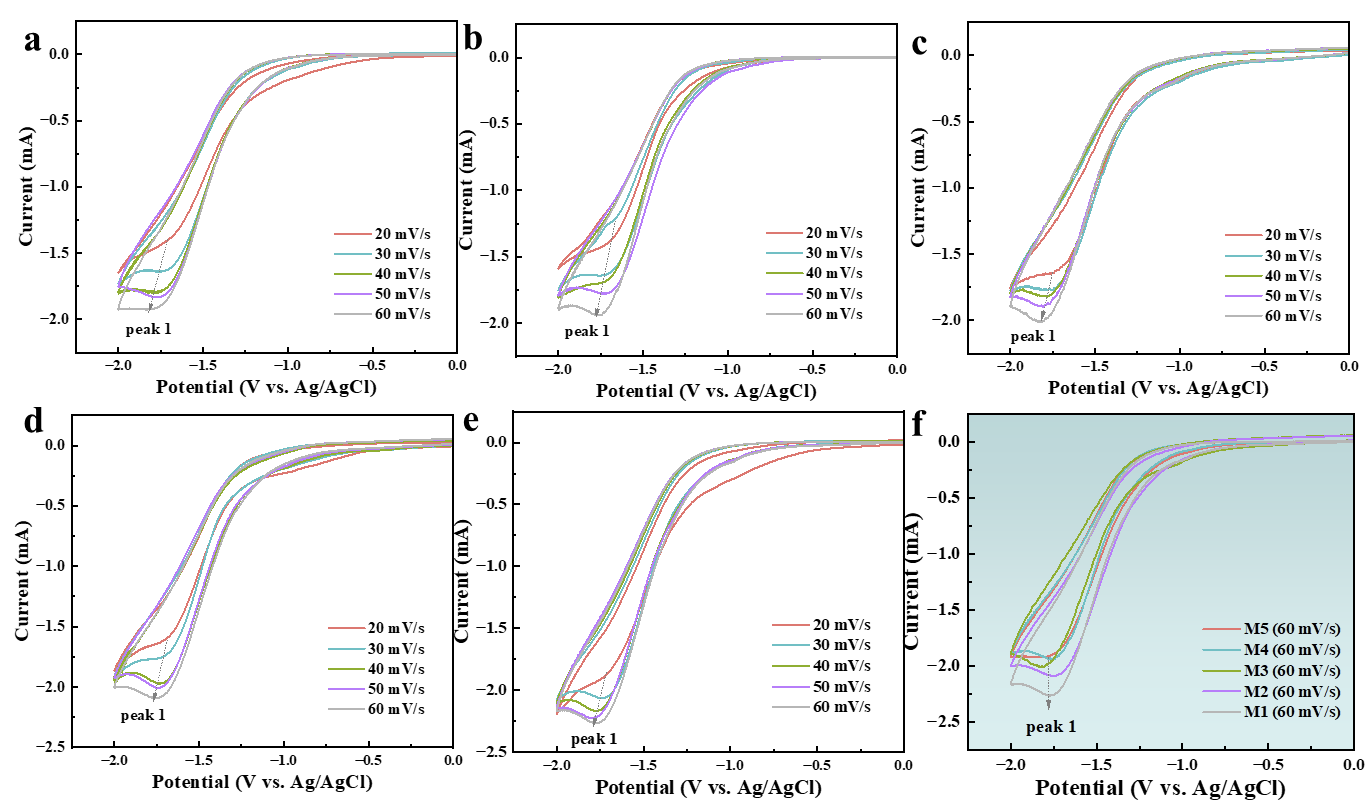


**Figure S8. (a-e)** CV curves of M5-4-3-2-1, which is adhered to indium tin oxide (ITO) using conductive adhesive, at scan rates ranging from 20 to 60 mV s^-1^. As the scan rate increases, the shape of the curve remains consistent, exhibiting a pair of oxidation-reduction peaks. These peaks are associated with the insertion and extraction processes of ions within the material's pores, demonstrating the stability of the electrochemical performance of the tetrahedral boron-based covalent organic framework (TB-COF). Additionally, **(f)** presents the CV curve of M5-4-3-2-1 at a scan rate of 60 mV s^-1^. As the film thickness gradually decreases, the capacitance increases, attributed to the shorter and less obstructed transport path for M1 ions, which facilitates the movement of both ions and electrons.

**Figure S9.** The current density and output power of the M1-5 membrane vary with load resistance. One side is placed in a dilute solution of 0.01 M NaCl, while the other side is immersed in a concentrated solution of 0.5 M NaCl. The output power of the M2 film reaches its maximum value at a resistance of approximately 10 kΩ. The output power, resistance, and current density of the other films are all lower than those of M2.


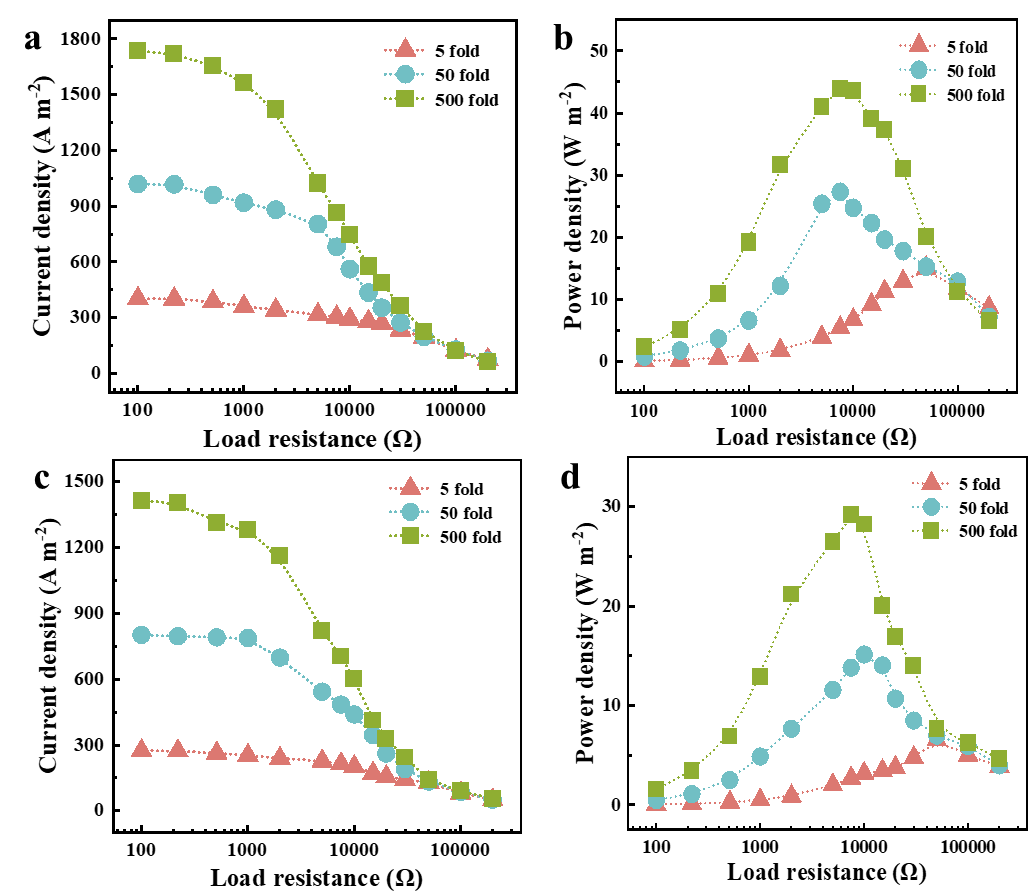


**Figure S10.** In the preferred experiment, a control experiment was conducted with multiple concentration gradient changes. The current density and maximum output power density of M2 and M4 at 5-fold, 50-fold, and 500-fold salt concentrations increase with the salinity gradient. Additionally, the output current density and maximum power of both M2 and M4 are further enhanced. Moreover, M2 demonstrates superior performance, exhibiting higher values than M4.


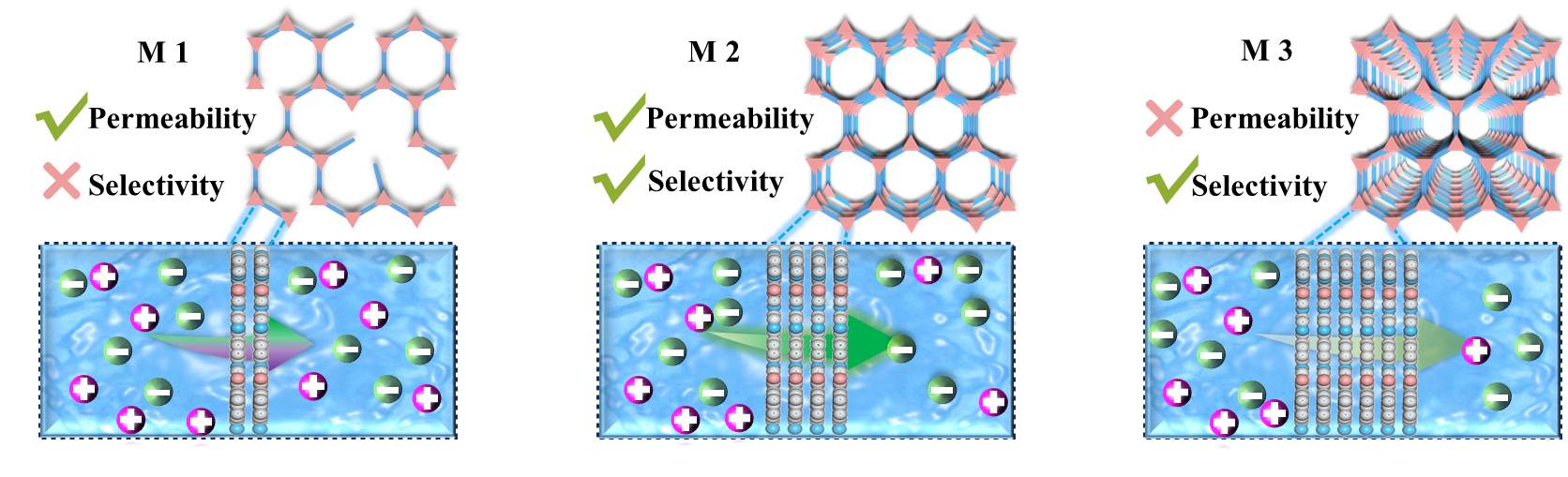


**Figure S11.** In molecular dynamics (MD) simulations the behavior of ions passing through the membrane was analyzed in terms of ion permeability and selectivity (M1-3). M1, due to its membrane defects, exhibited reduced ion selectivity. In contrast, the M2 structure maintains an ordered framework and appropriate ion channel length, resulting in excellent ion selectivity and permeability. The increased ion transport pathways in M3 have, to some extent, diminished ion permeability.


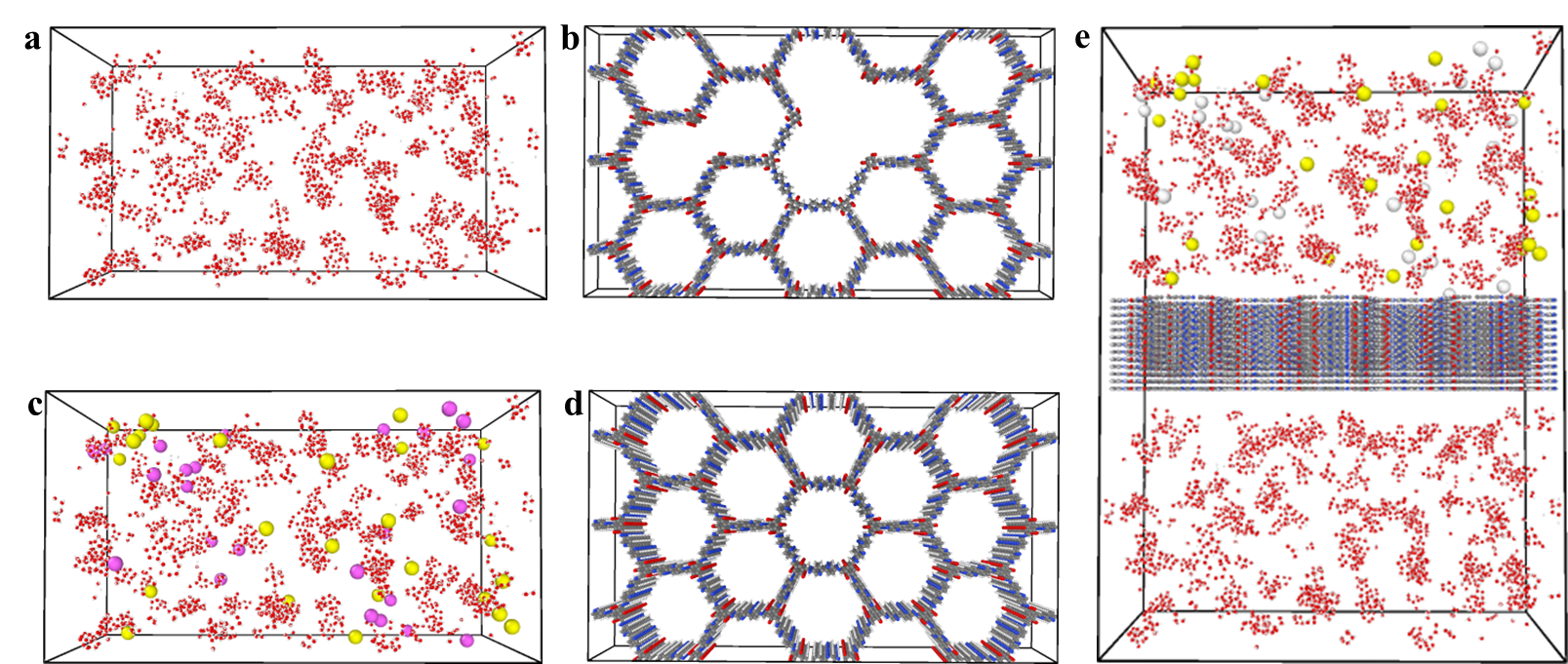


**Figure S12.** Basic information on parameter settings for molecular dynamics simulation process. By using Ovito software to view atomic trajectories and construct primitives, the basic data provided here are as follows: **(a)** The SPC water molecule model (2000 water molecules) shown in the simulation system; **(b)** The TB-COF structure of the defect corresponds to the structure of M1 in the simulation results; **(c)** NaCl aqueous solution model (2000 water molecules, 25 Na^+^ and 25 Cl^-^ ions); **(d)** The pristine TB-COF structure, corresponding to the complete ion channels (M2 and M3) in the simulation results; **(e)** The complete simulation system used for studying ion selectivity and permeability.


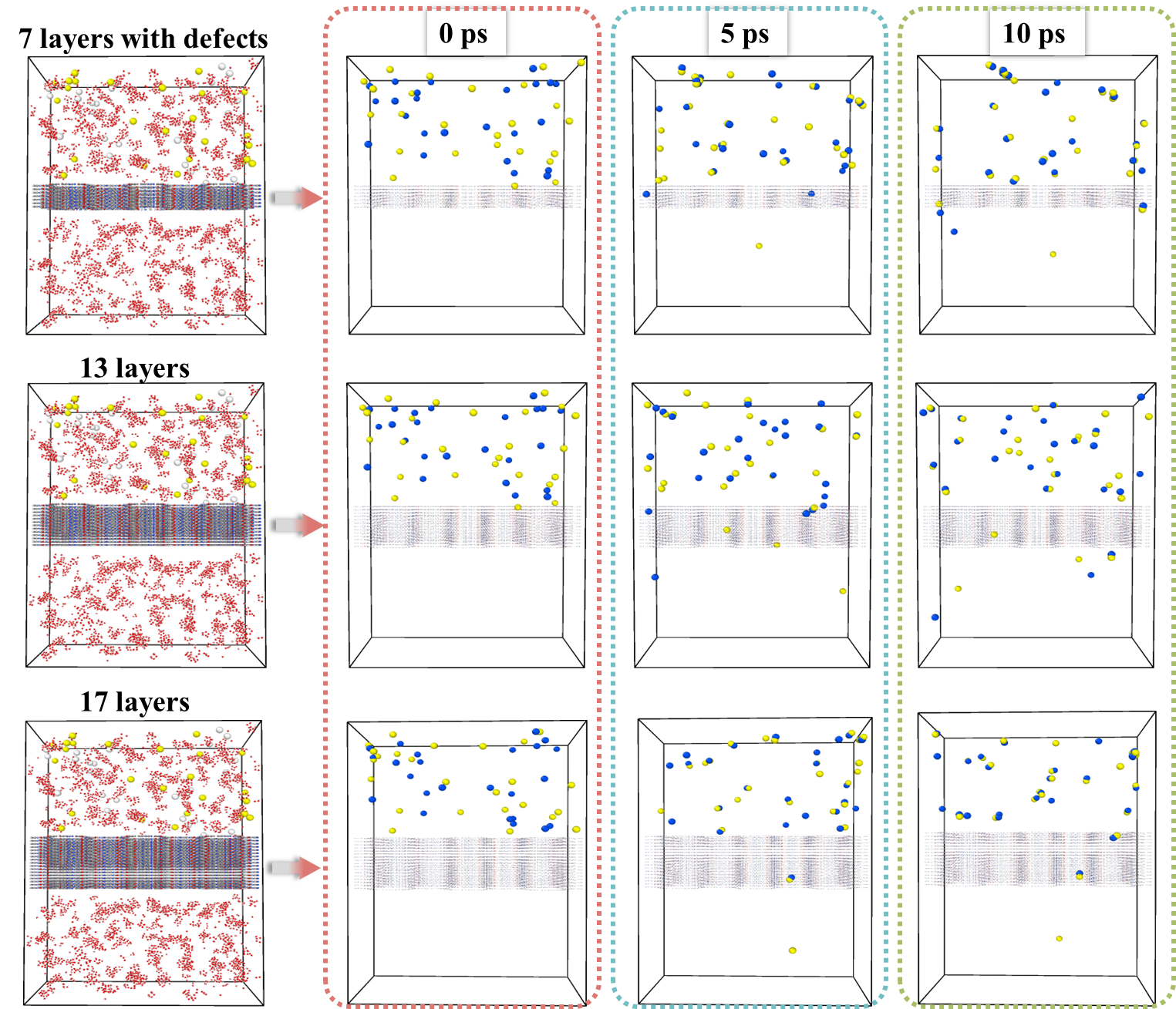


**Figure S13.** Snapshots from MD simulations illustrating the transmembrane transport of Na⁺ (blue) and Cl⁻ (yellow) through a TB-COF membrane. The COF structures were selected through an optimization process, resulting in the identification of three configurations: M1 (7 layers with defects), M2 (13 layers), and M3 (17 layers). Ion transport behaviors were compared within the time frame of 0–10 ps to evaluate the influence of membrane structure on ion selectivity. Notably, M2 demonstrated exceptional ion selectivity and permeability at 10 ps.


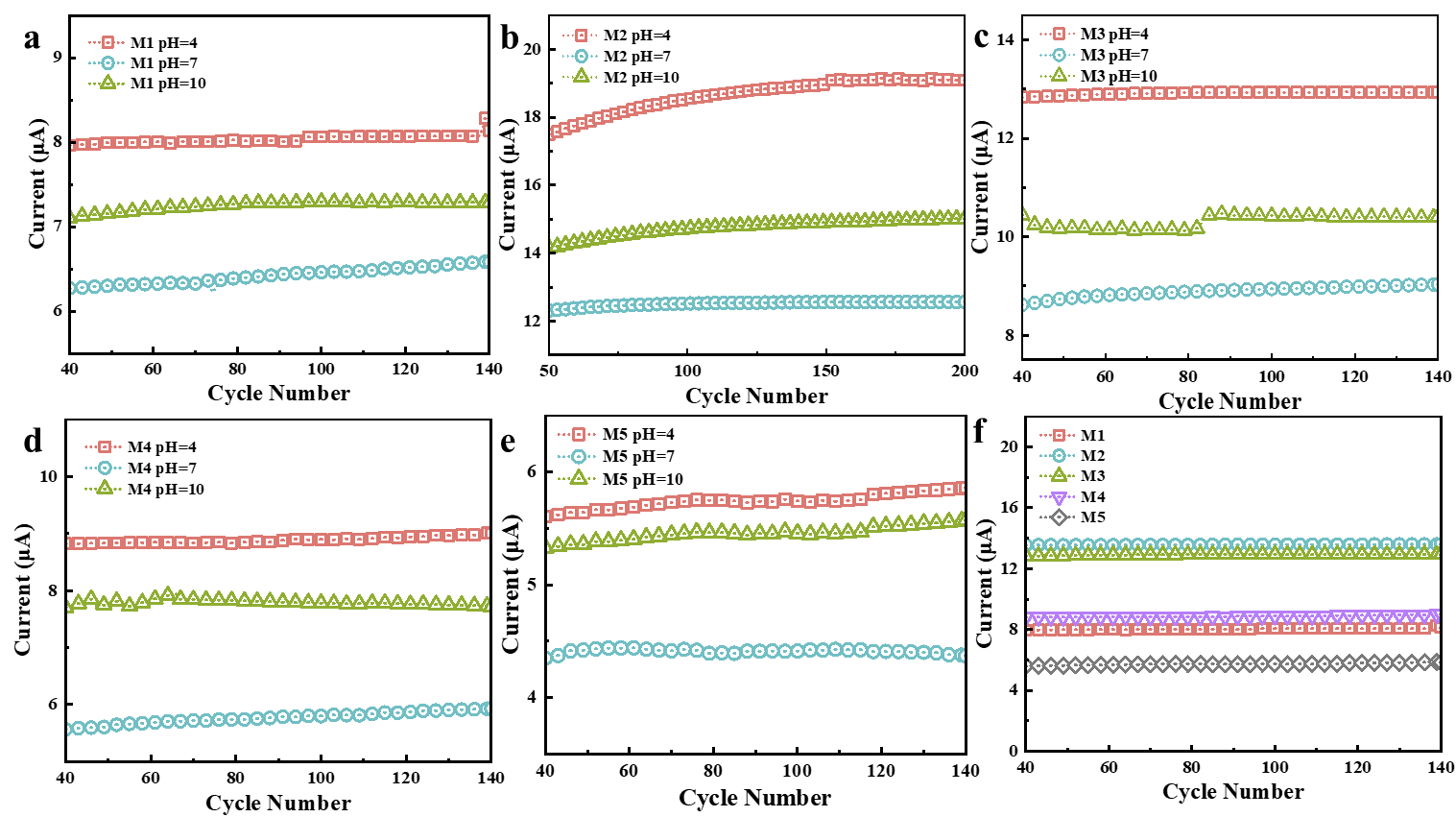


**Figure S14. (a-e)** When the two electrolytic cells contain 0.01 M KCl, the output current (SWEEP Cycle Number) of M1-2-3-4-5 at 2 V under pH conditions of 4, 7, and 10 achieves a stable and continuous output. M2 continues to demonstrate high ion transport performance and responsive results. **(f)** In summary, the ion current signal of M1-2-3-4-5 at pH=4, fixed at 2 V, indicates that M2 still exhibits excellent transmembrane ion transport performance.


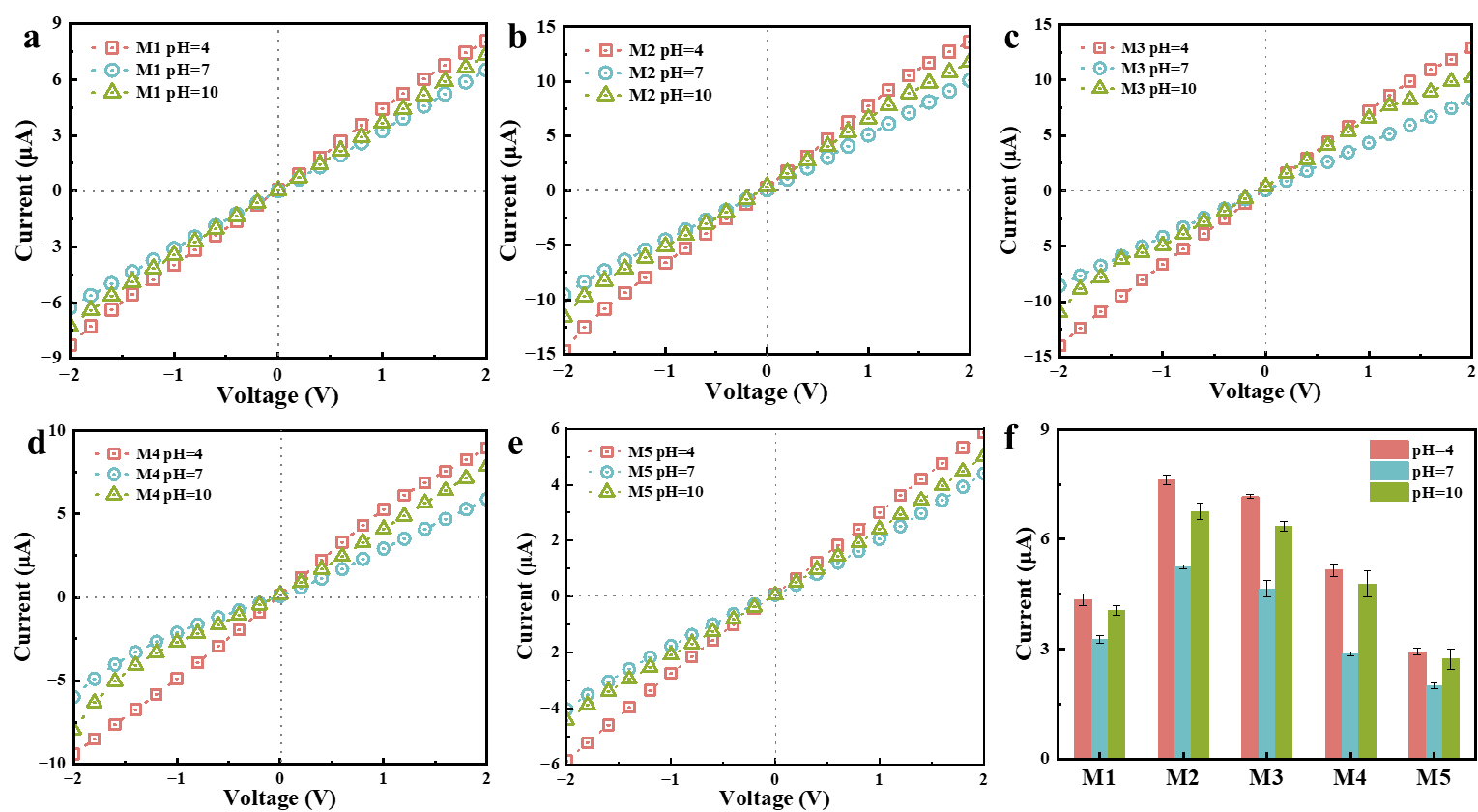


**Figure S15. (a-e)** The ion transport performance of M1-2-3-4-5 was evaluated under pH conditions of 4, 7, and 10, using two electrolytic cells containing 0.01 M KCl. M2 demonstrated sustained high ion transport performance and consistent response results. Additionally, the working membrane retained a uniform symmetrical structure across varying acid-base conditions. **(f)** The current values at a voltage of 1 V were consistent with the findings from the film thickness optimization experiment.


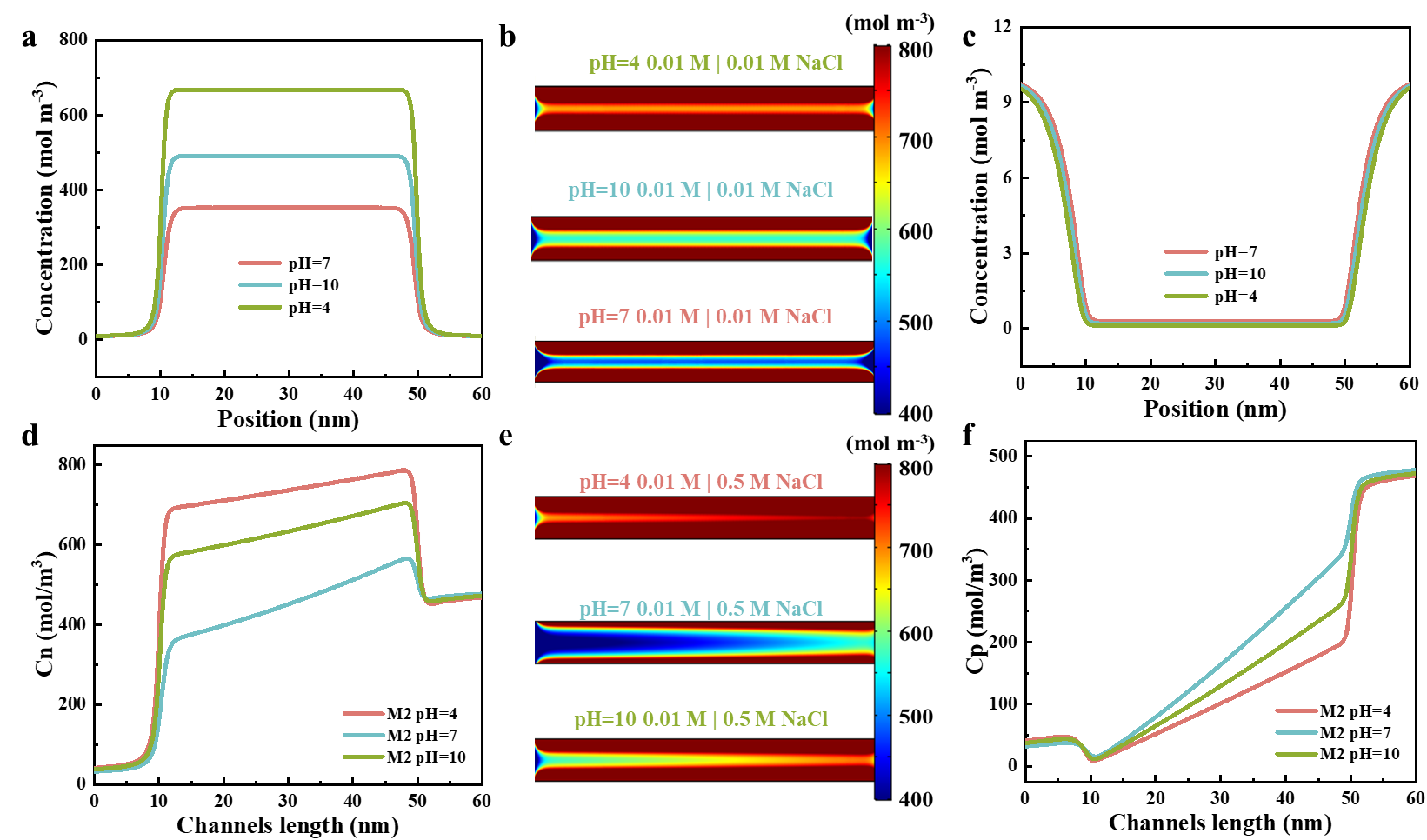


**Figure S16.** The simulation results of the charge density and pore size inside the nanochannel that vary with pH indicate that under the same pore size conditions, the distribution of electrolytes with the same concentration in the two electrolytic cells **(a), (b),** and **(c)**; Under 50-fold salinity gradient conditions **(d), (e),** and **(f)**, high charge density would contribute to improving ionic current density The simulated distribution shows the ion distribution inside the nano ion channel in a series of pH environments, which also confirms the ultra-high transmembrane ion transport performance under pH=4 conditions.


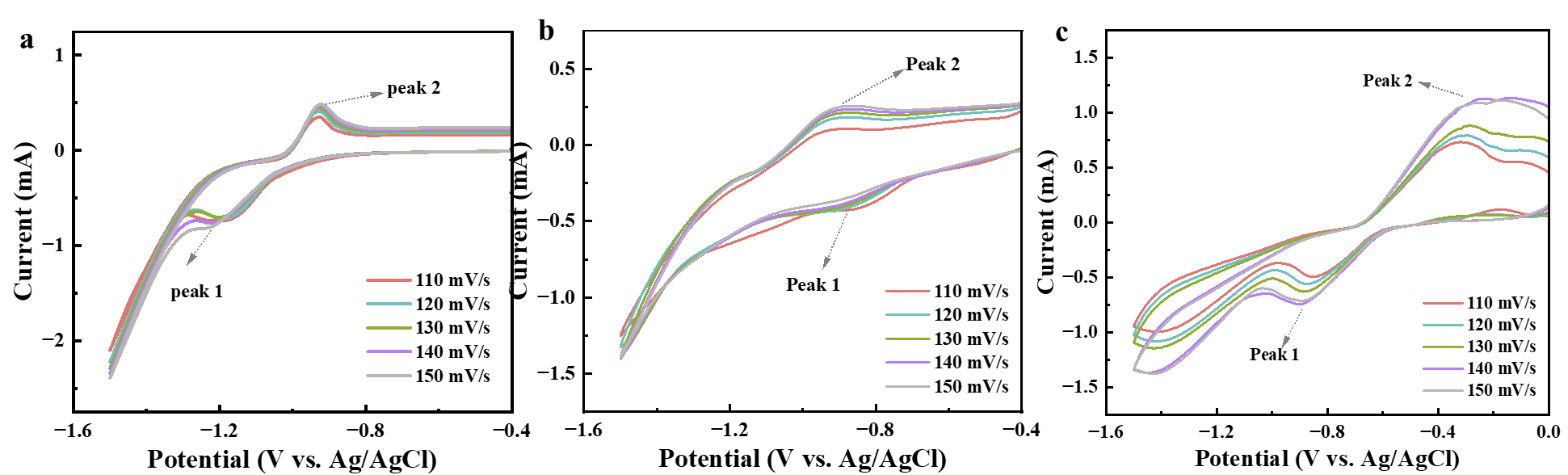


**Figure S17.** CV curves of M2 were obtained at various scanning rates of 110 mV s^-1^, 120 mV s^-1^, 130 mV s^-1^, 140 mV s^-1^, and 150 mV s^-1^ under the following conditions: **(a)** 0.01 M KCl, **(b)** 0.01 M KCl at pH 10, and **(c)** 0.01 M KCl at pH 4. As the scanning rate increases, the shape of the curves remains consistent, revealing a pair of oxidation-reduction peaks at approximately -0.9 V and -1.2 V in 0.01 M KCl, at -0.9 V in 0.01 M KCl at pH 10, and at -0.3 V and -0.9 V in 0.01 M KCl at pH 4. These peaks are associated with the insertion and extraction processes of ions within the material's pores. Additionally, the results demonstrate the stability of the electrochemical performance of TB-COF (M2) and indicate its effective ion insertion and extraction capabilities (high charge density) under pH=4 conditions.

**Figure S18.** TB-COF (a) shows the relationship between peak current density and scan rate^1/2^ at pH 4, 7, and 10.

According to the Randles-Sevcik equation, the diffusion coefficient of Cl^-^ in the membrane can be described as:

$$\begin{aligned} \boldsymbol{i}_{\boldsymbol{p}}\boldsymbol{=}\boldsymbol{kn}^{\boldsymbol{3/2}}\boldsymbol{A}\boldsymbol{D}^{\boldsymbol{1/2}}\boldsymbol{c}\boldsymbol{v}^{\boldsymbol{1/2}}\boldsymbol{\#}\left( \boldsymbol{7} \right) \end{aligned}$$

The peak current (*i_p_*) of the membrane is proportional to the square root of the scan rate (*v^1/2^*), and its slope is given by *kn^3/2^AD^1/2^c*. Note that this slope shows a positive correlation with the diffusion coefficient (*D*), as shown in **Figure S18.**

PH dependent diffusion as shown in **Figure S18**. The slope S (and thus D) was highest at pH 4, indicating that Cl⁻ diffuses more rapidly under acidic conditions. This is consistent with the protonation of bipyridine groups in the COF channels at low pH, which increases the positive surface charge density and enhances anion‑selective transport. The stable, reproducible CV responses across scan rates confirm the robust electrochemical stability of the TB‑COF membrane in acidic media.


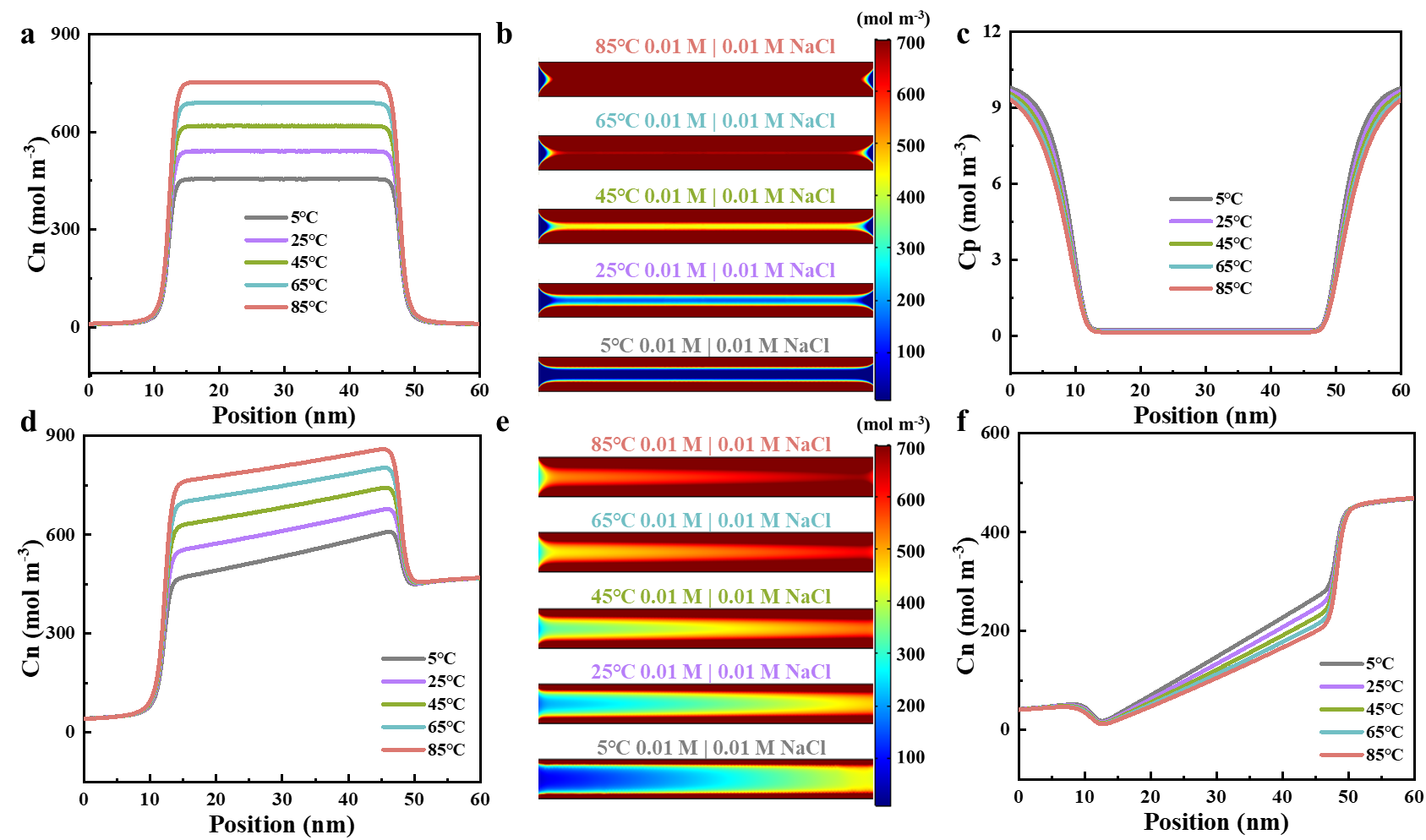


**Figure S19.** The simulation results of the charge density and pore size inside the nanochannel that vary with temperature indicate that under the same pore size conditions, the distribution of electrolytes **(a), (b),** and **(c)** with the same concentration in the two electrolytic cells is the same; Under the conditions of 50-fold salinity gradient **(d), (e),** and **(f)**, high charge density would contribute to improving ionic current density. The simulated distribution shows the ion distribution inside the nano ion channel under different temperature environments, which also confirms the ultra-high transmembrane ion transport performance at 85℃.


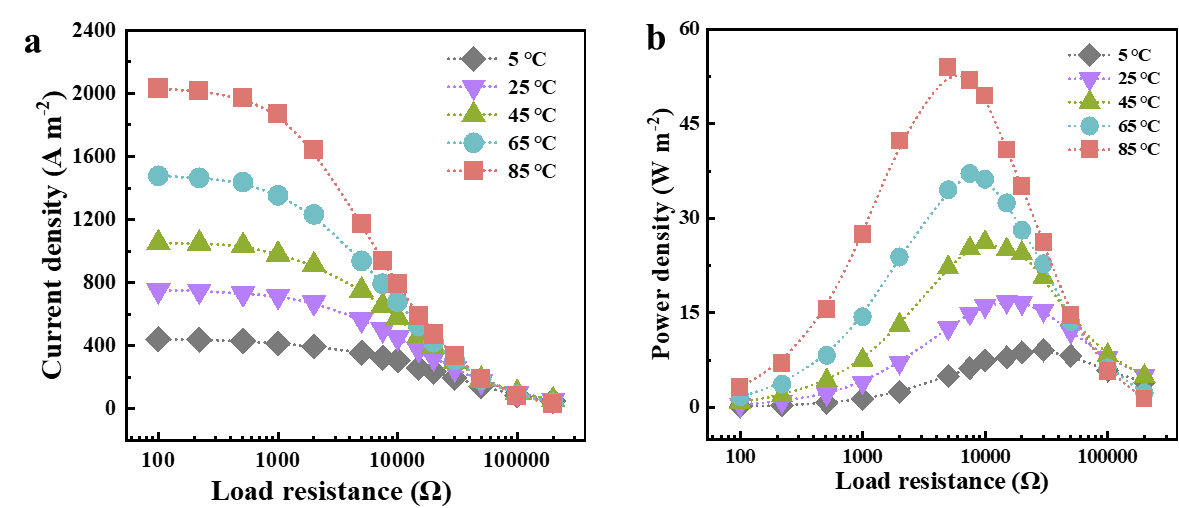


**Figure S20. (a)** and **(b)** respectively illustrate the current density and maximum output power density of M4 under various temperature conditions during the optimization experiment process. The temperature response test (universality) of the COF-4 (M4) film was conducted in environments at 5°C, 25°C, 45°C, 65°C, and 85°C, with corresponding power densities of 5.14 W m^-^², 16.03 W m^-^², 26.77 W m^-^², 33.58 W m^-^² and 65.45 W m^-^², respectively. The trend in current density changes was consistent with power generation. This consistency can be attributed to the fact that the migration rate of ions did not increase with the gradual rise in temperature, which ultimately resulted in an increase in efficiency.


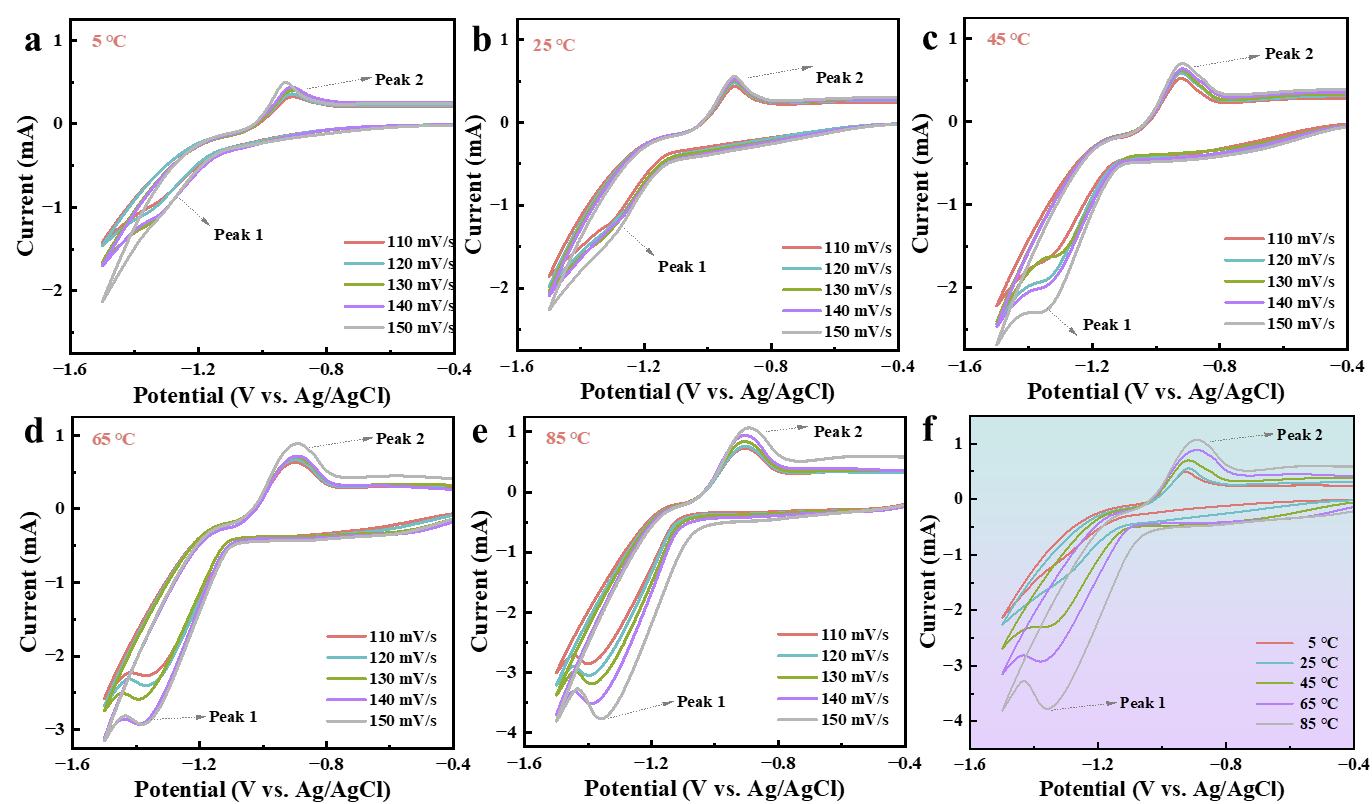


**Figure S21. (a), (b), (c), (d),** and **(e)** represent the CV curves of M2 at scan rates ranging from 110 to 150 mV/s at temperatures of 5°C, 25°C, 45°C, 65°C, and 85°C, respectively. As the scan rate increases, the shape of the curve remains consistent, exhibiting a pair of redox peaks associated with the insertion and extraction processes of ions within its pores. Variations in temperature influence the efficiency of these processes and also reflect the stability of the electrochemical performance of TB-COF. **(f)** displays the CV curves of M2 at a scan rate of 150 mV s^-1^ across the same temperature range. As the testing temperature rises, the migration rate of ions increases, leading to a corresponding rise in capacitance. Consequently, the presence of a certain amount of low-grade heat enhances the transport of ions and electrons, thereby facilitating more efficient energy conversion and collection.

**Figure S22.** The relationship between peak current density and scanning rate^1/2^ at 5℃, 25℃, 45℃, 65℃, and 85℃.

According to the Randles-Sevcik equation, the diffusion coefficient of Cl^-^ in the membrane. Temperature‑Dependent diffusion as shown in **Figure S22.** The derived D values followed the order: 85 °C > 65 °C > 45 °C > 25 °C > 5 °C. This trend reflects the accelerated ion mobility at elevated temperatures, attributable to reduced solution viscosity and weakened ion‑hydration shells. The result validates that low‑grade thermal energy can effectively boost ion transport and, consequently, osmotic energy conversion.


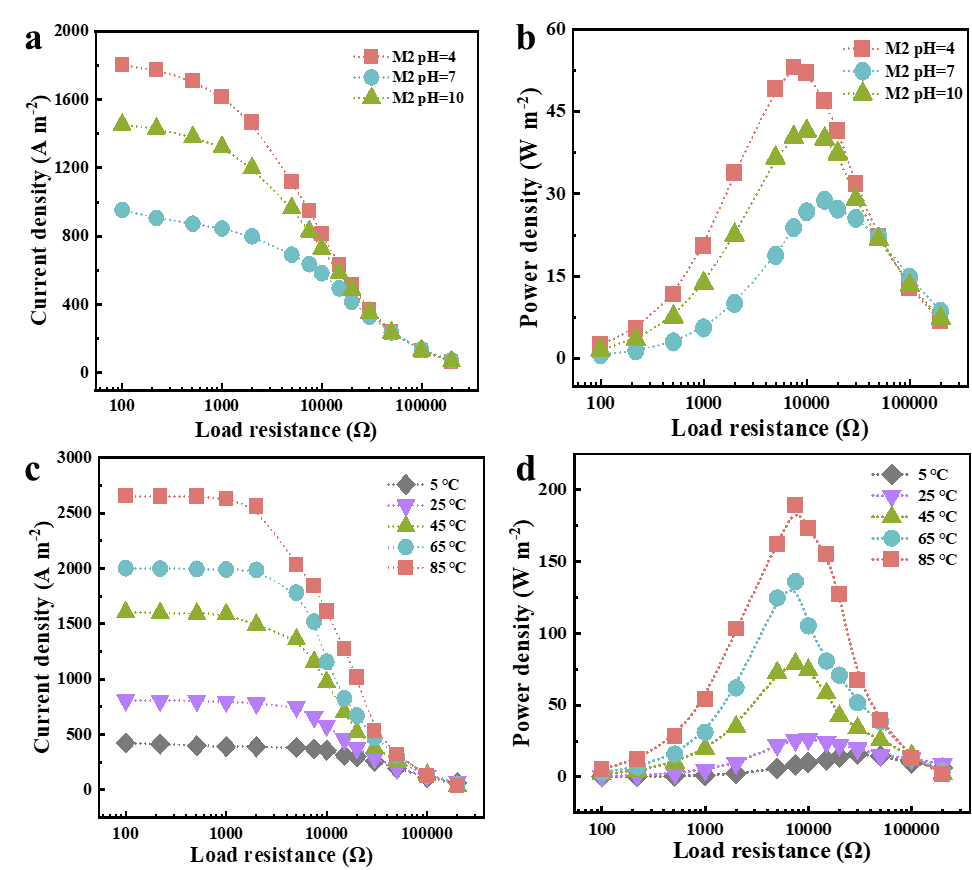


**Figure S23. (a) (b)** The output power and current density of M2 under a series of external loads were analyzed. As the load resistance gradually increases, the current density decreases, and the output power reaches its maximum value at approximately 15, 10, and 7.5 kΩ, respectively. Notably, at pH=4, the maximum output power density was achieved. **(c) (d)** The output power and current density of M2 were also examined under varying ambient temperatures. As the ambient temperature increases, the current density correspondingly rises. At 85°C, the output power reaches its maximum value at around 10 kΩ, resulting in a maximum output power density of 190.52 W m^-^².


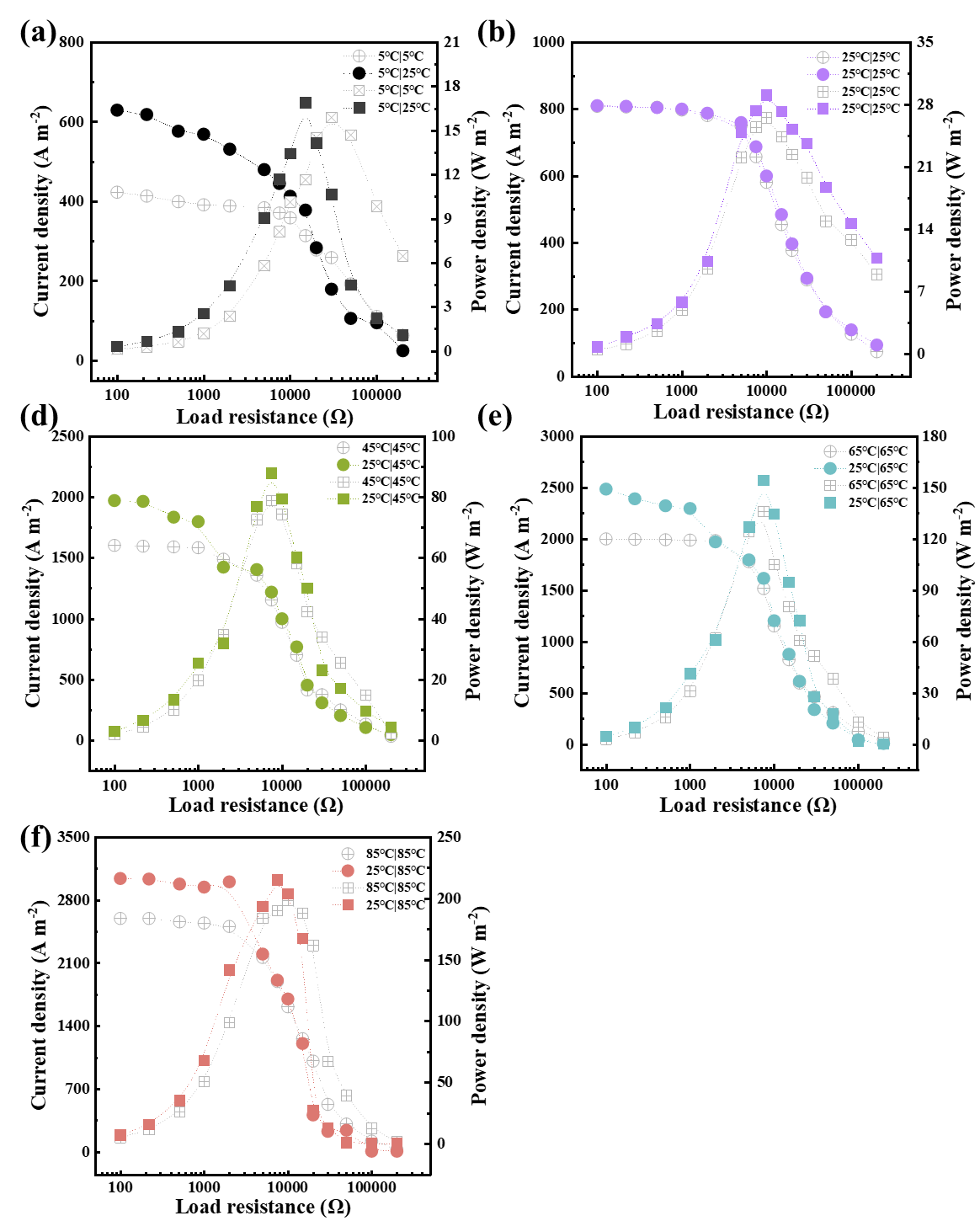


**Figure** **S24.** (a-e) Comparison of current density and power density corresponding to symmetric temperature fields (5°C | 5°C, 25°C | 25°C, 45°C | 45°C, 65°C | 65°C and 85°C | 85°C) and asymmetric temperature fields (5°C | 25°C, 25°C | 45°C, 25°C | 65°C and 25°C | 85°C) under a 50-fold salinity gradient.

To address your valid concern and strengthen the practical relevance of our study, we have conducted additional experiments under an asymmetric thermal field. As shown in the **Figure S24**, we tested the system under a 50-fold salinity gradient with the following temperature differentials (ΔT) across the membrane: 5°C | 25°C, 25°C | 25°C (symmetric, as a baseline), 25°C | 45°C, 25°C | 65°C, and 25°C | 85°C.

**Figure** **S25.** The summary of output power density under different transmembrane ΔT intuitively reflects the enhancing effect of temperature gradient on energy conversion.

As shown in the **Figure S25**, Under the largest ΔT of 60°C (25°C | 85°C), the output power density reached 215.00 W m^-2^, representing an 11.39% enhancement compared to the performance under an equivalent symmetric high-temperature condition. This improvement is attributed to the significant thermal-osmotic effect induced by the asymmetric thermal field. A larger ΔT promotes ion thermophoretic mobility more effectively, which notably mitigates adverse effects like concentration polarization. Consequently, the TB-COF membrane system, driven by the combined salinity and temperature gradients, achieves superior transmembrane ion transport and enhanced energy conversion efficiency.


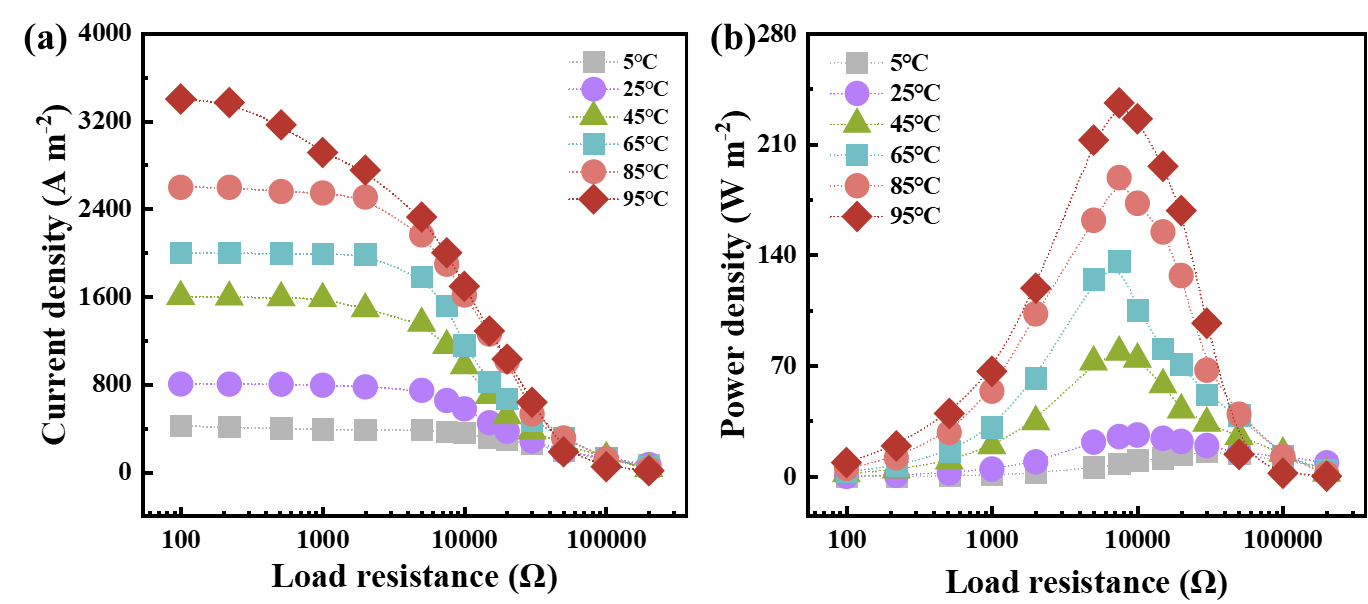


**Figure** **S26.** (a) (b) The output power and current density of TB-COF were examined under varying ambient temperatures. As the ambient temperature increases, the current density correspondingly rises. At 95°C, the output power reaches its maximum value at around 10 kΩ, with an output current density of 3403 A m^-2^ and a maximum output power density of 236.27 W m^-2^.

Measure the current density and output power density values at 95°C under a 50-fold salinity gradient (0.01 M | 0.5 M NaCl), as shown in **Figure S26**. As expected, the output power density does continue to increase with increasing temperature. At 95°C, the output power density reached 236.27 W m^-2^ and the current density was 3403 A m^-2^, further setting a new record for permeation energy conversion membranes at low temperatures.


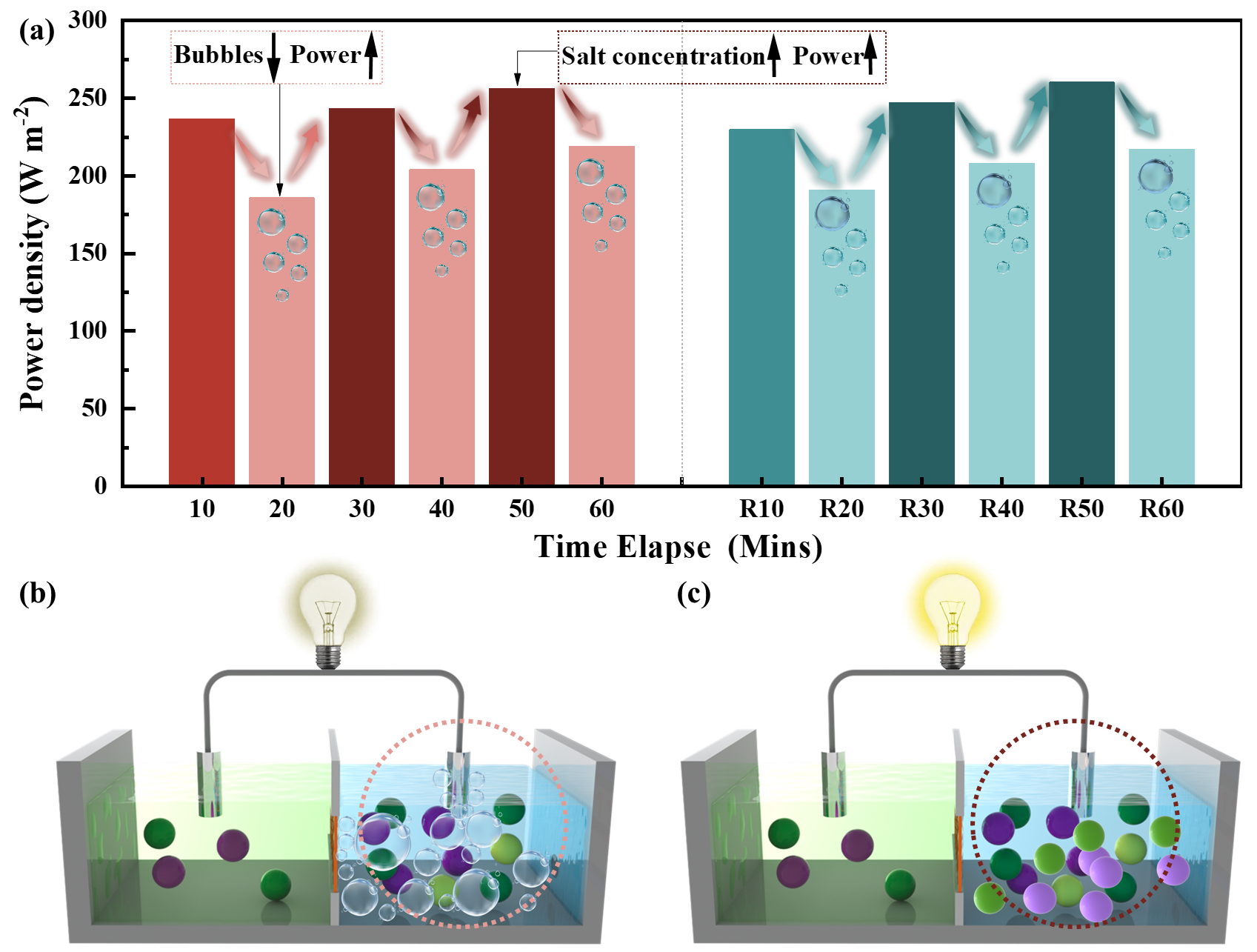


**Figure S27. Short term stability test of TB-COF film output power density at high temperature of 95°C.** (a) The experiment was conducted for 1 hour under a 50-fold salinity gradient (0.01 M | 0.5 M NaCl) and recorded every 10 minutes. The red data points represent the initial instantaneous power density; The light red range indicates the accumulation of bubbles in the electrolytic cell and the damage to the membrane electrolyte interface contact. The deep red range indicates that after manually removing bubbles, the power increases with the increase of salt concentration. The blue interval represents the second cycle test (R10-R60 minutes) conducted after membrane activation regeneration, and its trend of change is basically consistent with the first cycle, indicating that the output fluctuations at high temperatures are mainly caused by interface bubbles and concentration disturbances, and the performance of the membrane itself can be restored through regeneration. (b) During continuous testing at 95℃, the electrode surface and membrane surface were covered by bubbles, which hindered mass transfer and resulted in a decrease in power. (c) After manually removing the bubbles, as the water evaporates, the electrolyte concentration increases, the conductivity increases, and the power also increases.

At 95°C, the violent evaporation of local water and the generation of bubbles can disrupt the stability of the membrane electrolyte interface (corresponding to the light pink and light blue ranges in **Figure S27a**), and the power has been reduced to ~185 W m^-2^. After removing the interference of bubbles, the evaporation of water over time interferes with the uniformity of electrolyte concentration (corresponding to the deep pink and deep blue ranges in **Figure S27a**), and the power has rebounded to ~243 W m^-2^. The interference between bubbles and salt concentration observed in the above experiment is shown in **Figure S27b and c**. In the experiment, continuous manual removal of bubbles is required, which not only leads to measurement errors but also brings uncertainty to the operation of the equipment. Although the temperature of many low-grade heats does not exceed 100°C and the structure of TB-COF materials remains stable after boiling water treatment, interface and liquid phase stability issues still need to be addressed at temperatures close to boiling.


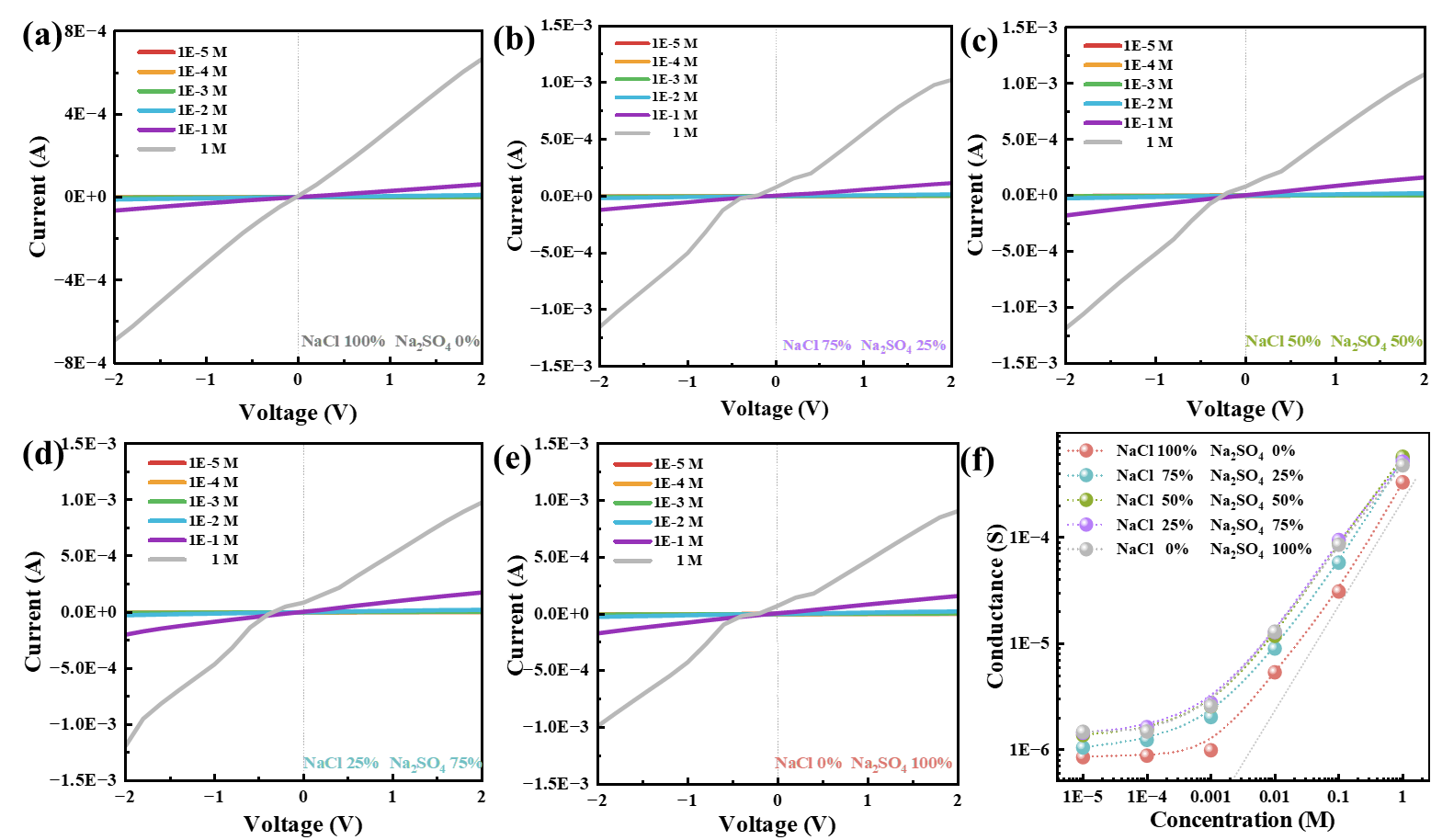


**Figure** **S28. Ionic transmembrane transport performance of TB-COF membrane at different SO₄²⁻ concentrations.** (a–e) The I-V curves of NaCl/ Na_2_SO₄, mixed solutions with different SO₄²⁻ contents (0%, 25%, 50%, 75%, and 100%) (concentration gradient: 10⁻⁵~1 M) at -2 V to 2 V voltage. (f) Ionic conductivity of TB-COF membrane in different concentrations of NaCl, Na_2_SO₄, and their mixed solutions at 1 V voltage.

We found that as the proportion of SO₄²⁻ in the salt solution gradually increases **(Figure S28a-e**), the obtained I-V curve is nonlinear and does not cross the origin. This is mainly attributed to the much higher binding energy between SO₄²⁻ and COF nanochannels compared to Cl⁻. SO₄²⁻ is more easily adsorbed by the membrane on the inner wall of the channel, resulting in a lower diffusion coefficient of SO₄²⁻ in the nanofluid channel than Cl⁻, leading to heterogeneity in the nanofluid channel. Furthermore, our data on concentration-dependent selectivity reveal that the Cl⁻/SO₄²⁻ selectivity of the membrane increases at higher salt concentrations **(Figure S28f)**. A plausible explanation is that at high concentrations, SO₄²⁻ ions may accumulate near channel entrances or form clusters, partially impeding efficient nanochannel operation. This observation is consistent with the following concerns, mainly due to the high charge and large hydration radius of SO₄²⁻, which increase the transmission resistance.


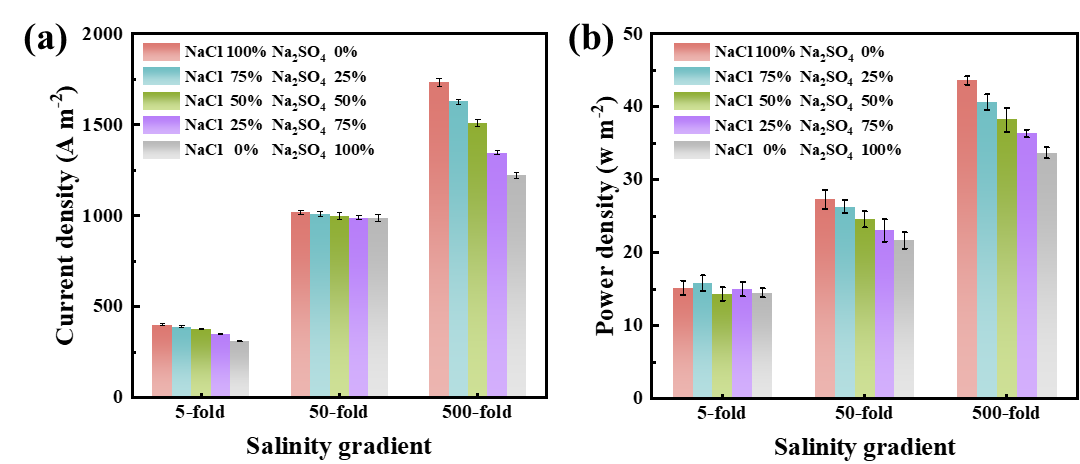


**Figure** **S29. Permeation energy conversion performance of TB-COF membrane under different SO₄²⁻ contents.** (a, b) Comparison of current density and power density with different SO₄²⁻ contents (0%, 25%, 50%, 75%, 100%) at the same salinity gradient (5-fold 50-fold, and 500-fold).

Under a 500-fold salinity gradient, the current density decreased from 1732.98 A m^-2^ to 1221.31 A m^-2^, and the power density decreased from 43.60 W m^-2^ to 34.98 W m^-2^, a decrease of 19.77%. This is attributed to the strong binding energy between SO₄²⁻ and the inner wall of the pore, which makes it easy to be adsorbed and retained, thereby affecting its own and other ion diffusion. Under high concentration conditions, ions are more likely to aggregate at the entrance or inside the pore, further hindering transport and even causing some nanochannels to be difficult to effectively utilize.


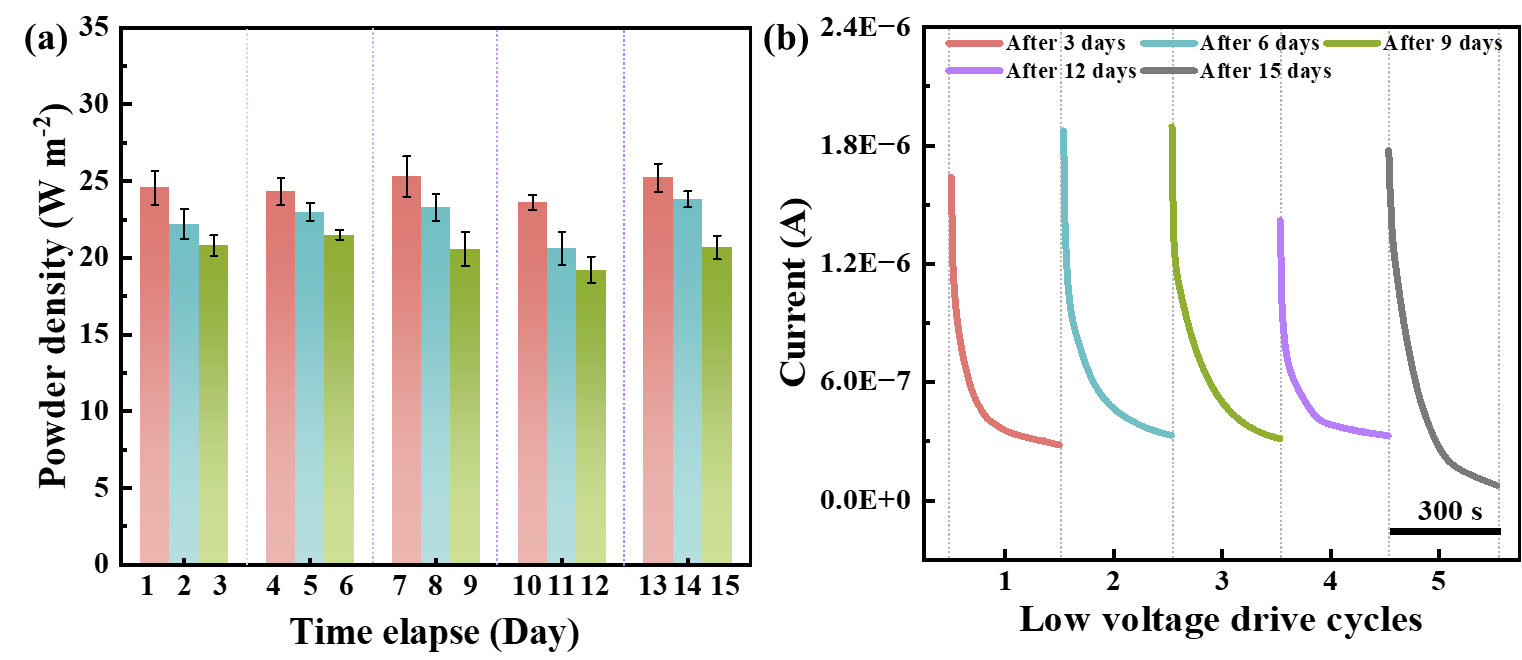


**Figure R9-** **S30. Study on the anti-pollution performance and renewability of TB-COF membrane.** (a) Conduct 5 consecutive cycles of pollution regeneration testing in a mixed solution system of 0.01 M | 0.5 M NaCl, Na₂SO₄, MgCl₂, and MgSO₄, with each cycle consisting of 3 days of continuous operation for a total of 15 days, monitoring changes in power density. (b) After each pollution stage, the membrane is briefly soaked and cleaned, and then the current time (I-T) curve is tested for 300 seconds at a voltage of 1 V to evaluate the electrochemical regeneration effect of the membrane.

In actual desulfurization wastewater systems, the pollution and scaling risks caused by specific components such as high concentrations of sulfates and magnesium salts are more severe. How to alleviate the deposition and blockage of such ions in membrane pores is crucial for maintaining long-term operational stability. To this end, we further proposed and validated an antipollution strategy based on waste acid environment and low voltage drive. By utilizing the acidic conditions of desulfurization wastewater and applying a lower transmembrane voltage, it is possible to effectively promote the directional migration of pollutants such as sulfate ions and magnesium ions in the nanochannels, thereby accelerating their detachment from the membrane pores and avoiding continuous accumulation and scaling. This method not only effectively alleviates membrane fouling caused by specific ions, but also significantly reduces the secondary pollution that traditional chemical cleaning may cause, providing a clean and efficient solution for membrane maintenance in actual wastewater treatment processes.

To further evaluate the anti-pollution ability and renewability of TB-COF membrane in practical complex systems, we simulated the ion composition of desulfurization wastewater and prepared a mixed electrolyte solution containing NaCl, Na₂SO₄, MgCl₂, and MgSO₄. We conducted a 3-day continuous permeation energy collection test under a 50-fold salinity gradient, and then applied low voltage to drive membrane regeneration on the 4th day, and repeated performance monitoring, completing a total of 5 cycles **(Figure S30a)**. The results indicate that complex ion systems can have a reversible effect on membrane performance: in the first cycle, the output power density gradually decreased from the initial 25.67 W m^-2^ to 20.8 W m^-2^ on the third day, with a decrease of about 18.97%. This decline is mainly due to the competitive transport and transient adsorption of multiple ions within the nanochannel, which affects the selectivity and permeation efficiency of the channel in the short term. After each cycle, we use simple immersion cleaning to remove residual salt on the membrane surface, supplemented by low voltage (1 V) directional driving **(Figure S30b)**. This treatment can effectively promote the desorption of ions trapped in the channel, reducing the membrane output current from about 1.5 μA to 0.3 μA within 300 seconds. This value is even lower than the conductivity level of the membrane in a 10-5 M NaCl solution, indicating effective removal of contaminated ions in the channel. The performance decline trend in the following four cycles is basically consistent with the first cycle, indicating that the cleaning, soaking, and regeneration methods have stable recovery effects. The above membrane regeneration method is also the method used in the original manuscript to record long-term stability.

**Supporting Table**

**Table S1.** Preparation conditions during optimization of TB-COF membranes.

| **Membranes** | **Tp**  **(mg, mmol)** | **Bpy**  **(mg, mmol)** | **PTSA**  **(mg, mmol)** | **HAc**  **(μL)** |
| --- | --- | --- | --- | --- |
| M1 | 2.10, 0.01 | 2.80, 0.02 | 5.70, 0.03 | 10 |
| M3 | 8.40, 0.04 | 11.20, 0.06 | 22.80, 0.12 | 40 |
| M4 | 12.60, 0.06 | 16.80, 0.09 | 34.20, 0.18 | 60 |
| M5 | 16.80, 0.08 | 22.30,0.12 | 45.70, 0.24 | 80 |

**Table S2.** comparison of output power density between the TB-COF membrane and existing membrane materials.

| **Membranes** | **Temperature (℃)** | **Power Density (W m^-2^)** | **Refs** |
| --- | --- | --- | --- |
| COF-SO_3_Na | 80 | 231.00 | Ref.1b |
| GO | 40 | 9.87 | Ref.2a |
| Mxene-BN | 43 | 6.20 | Ref.4a |
| SPEEK/PES | 55 | 16.50 | Ref.4d |
| HOF | 50 | 96.80 | Ref.6a |
| AAO | 45 | 25.50 | Ref.6b |
| TC-LTC | 70 | 17.70 | Ref.28b |
| AFNs | 70 | 9.00 | Ref.31a |
| ABN | 95 | 0.60 | Ref.31b |
| COF-EB | 65 | 210.00 | Ref.31c |
| GDS | 45 | 306.10 | Ref.31d |
| **TB-COF** | **85** | **258.81** | **This work** |

**Supporting Video**

**Video S1** The motor propeller device is powered by a TB-COF membrane-based generator.
